# Compositional and Structural Modifications by Ion Beam in Graphene Oxide for Radiation Detection Studies

**DOI:** 10.3390/ijms232012563

**Published:** 2022-10-19

**Authors:** Mariapompea Cutroneo, Lorenzo Torrisi, Letteria Silipigni, Alena Michalcova, Vladimir Havranek, Anna Mackova, Petr Malinsky, Vasily Lavrentiev, Pavol Noga, Jozef Dobrovodsky, Petr Slepicka, Dominik Fajstavr, Lucio Andò, Vaclav Holy

**Affiliations:** 1 Nuclear Physics Institute AS CR, Hlavni 130, 250 68 Rez, Czech Republic; 2 Dipartimento di Scienze Matematiche e Informatiche, Scienze Fisiche e Scienze della Terra, 98166 Messina, Italy; 3 INFN Sections of Catania, S. Sofia 64, 95123 Catania, Italy; 4 Department of Metals and Corrosion Engineering, University of Chemistry and Technology, Technická 5, 166 28 Prague, Czech Republic; 5 Department of Physics, Faculty of Science, University of J. E. Purkyně, Pasterouva 3544/1, 400 96 Ústí nad Labem, Czech Republic; 6 Faculty of Materials Science and Technology in Trnava, Advanced Technologies Research Institute, Slovak University of Technology in Bratislava, Jána Bottu 25, 91724 Trnava, Slovakia; 7 Department of Solid State Engineering, University of Chemistry and Technology, 166 28 Prague, Czech Republic; 8 Department of Condensed Matter Physics, Faculty of Mathematics and Physics, Charles University, Ke Karlovu 5, 121 16 Praha, Czech Republic

**Keywords:** GO, dosimeter, ion beam irradiation, graphene quantum dots, CNT, AFM, SEM, TEM

## Abstract

In the present study, graphene oxide foils 10 μm thick have been irradiated in vacuum using same charge state (one charge state) ions, such as protons, helium and oxygen ions, at the same energies (3 MeV) and fluences (from 5 × 10^11^ ion/cm^2^ to 5 × 10^14^ ion/cm^2^). The structural changes generated by the ion energy deposition and investigated by X-ray diffraction have suggested the generation of new phases, as reduced GO, GO quantum dots and graphitic nanofibers, carbon nanotubes, amorphous carbon and stacked-cup carbon nanofibers. Further analyses, based on Rutherford Backscattering Spectrometry and Elastic Recoil Detection Analysis, have indicated a reduction of GO connected to the atomic number of implanted ions. The morphological changes in the ion irradiated GO foils have been monitored by Transmission Electron, Atomic Force and Scanning Electron microscopies. The present study aims to better structurally, compositionally and morphologically characterize the GO foils irradiated by different ions at the same conditions and at very low ion fluencies to validate the use of GO for radiation detection and propose it as a promising dosimeter. It has been observed that GO quantum dots are produced on the GO foil when it is irradiated by proton, helium and oxygen ions and their number increases with the atomic number of beam gaseous ion.

## 1. Introduction

Although Wallace [1] explored graphene theory in 1947, only in 1994 was the term ‘graphene’ introduced for the first time [2]. It derives from the fusion of graphite and the suffix–ene, which in inorganic chemistry indicates a one-atom thick two-dimensional layer of atoms. Over the family of graphene-based materials, graphene oxide arises as precursor of graphene for its cost-effective large-scale synthesis. Dating back to the 1840s [3], graphene oxide consists of sp2 and sp3 bonded carbon atoms, which can be envisioned as a graphene layer covered on both sides by different oxygen functional groups such as epoxide (-C-O-C), carbonyl (C=O), carboxyl (O=C–OH), hydroxyl (-OH), phenol groups, water and adsorbed gases (O_2_, H_2_).

GO is realized by oxidation and then exfoliation of graphite. The GO features are strongly affected by differences in the starting graphite material as well as in the preparation methods and in the atmosphere conditions. The native insulator feature of GO can be turned in semiconductive/conductive removing the oxygen content and inducing the recovery of trigonally bonded sp2 carbon atoms. In this way reduced graphene oxide (rGO) is obtained. Liquid-phase chemical reduction by hydrazine (a strong reductant), thermal reduction in vacuum or inert atmosphere, ion irradiation [4] and laser irradiation [5] are a few of the reported approaches to turn GO into rGO. The flourishing applications involving GO include temperature [6] and pressure sensors [7], smart composites [8], microcapacitors [9,10], biomarkers [11] and cell culture scaffolds in nanomedicine [12,13].

A recent topic attracting attention is the design of biocompatible, hemocompatible and water equivalent foils to be used as small dosimeters for the validation of the dose for proton and carbon radiation therapies, and electrons and X-ray irradiation acquired in real-time.

An endless list of new dosimeter foils is now commercially available such as the radiochromic films (RCF), the CR39 solid state nuclear track detectors and the LiF-rich thermoluminescent sheet-type dosimeters [14]. These are tested thin dosimetric foils to be employed from low doses (cGy) up to high doses (hundreds MGy).

RCFs and CR39 are commonly employed for relative dosimetry in stacks to obtain quantitative information on the energy spectrum of a specific bunch of accelerated protons [15]. Different models of RCF are available, with different sensitivities to be used in different dose ranges and different depths of the sensitive component. The most recent versions of RCFs are the Gafchromics whose sensitivity to radiation depends on a solid solution of colorless crystalline diacetylene monomer (sensitive component) that coats a flexible film; when the active component is exposed to radiation, it reacts to form a blue colored polymer (self-developing films), with the possibility of handling in the visible light. The readout of the change in optical density following dose deposition in the Gafchromic films is usually carried out with transmission densitometers, film scanners, spectrophotometers or using flatbed RGB scanners in the red channel [16]. The Gafchromic films based on their high spatial resolution can be used to collect a precise measurement of 2D dose distributions at different depths: this is crucial for the cell irradiation. The CR-39 is a polycarbonate sheet used as detector of protons and heavy ions [17]. When a particle impinges on it, it produces molecular damage in the cylindrical region of the crossed material extended for tens of nano-meters along the particle trajectory (Latent Track). The following chemical etching transforms these damaged trails into permanent structures called ion tracks. The preferable media for ionizing radiation dosimetry measurements specially in clinical radiation oncology [18] should exhibit small energy dependence, high spatial resolution (for RCFs it is up to 50 µm) and tissue-equivalence.

Graphene oxide can be promising as sensitive material for small size dosimeters thanks to some of its features such as the low density, biocompatibility and tissue-equivalence. These small GO based dosimeters could provide a permanent reading and a 3D spatial distribution of the dose, crucial parameters in radiotherapy and radioactive contamination, just to name a few. In our previous works [19,20,21], the reliability of GO as a dosimeter was studied using different types of ions, energies and high fluences. It was observed that in regime of electronic stopping power the GO reduction was linearly dependent on the absorbed dose and when the nuclear stopping power was not negligible anymore, defects and amorphization of the material were induced and the linearity was lost.

Now the controllable modifications induced in the ion irradiated GO at low fluences are investigated to propose the GO as prospective dosimeter. In addition, following the results described by Olejniczak et al. [22], nanosized rGO spots can be created by swift heavy ion (SHI) bombardment of GO films. These rGO spots can be considered graphene QDTs embedded in a non-conducting matrix when SHIs with low and intermediate electronic stopping power Se values irradiate GO foils, whereas ions with high Se values can form coupled QDTs-antidots with sp-C bridge.

GQDTs, zero-dimensional photoluminescence (PL) carbon-based nanomaterial with mostly circular or elliptical shapes, consist of very thin (typically 3–20 nm) graphene sheets [23] and lateral dimensions less than 100 nm.

They usually contain functional groups (carboxyl, hydroxyl, carbonyl and epoxide) at their edges that can act as reaction sites and alter photoluminescence emission from the dots by changing their electron density [24].

Although the existing preparation methods of GQDTs [25] mainly focus on other approaches (i.e., the hydrothermal cutting and the oxidative cutting carbon fiber methods) which are relatively difficult and introduce several strong acids, the radiation synthesis technology appears a more simple, rapid, scaled and efficient preparation method of GQDTs. The electron or ion itself are strong reducing agents, so the extra chemical reductant does not need to be added during the synthesis processes. Moreover, the morphology and size could be adjusted by controlling the radiation dose, which provides a potential method for preparing customized-size GQDTs [26].

Based on this information, in this paper we report the realization of nanometer-sized graphene oxide spots at low doses of ion irradiation in the regime of electronic stopping power. These formed structures could be attractive for applications requiring large area coverage such as light controlled conductive switching [27].

## 2. Results and Discussion

**Figure 1:**
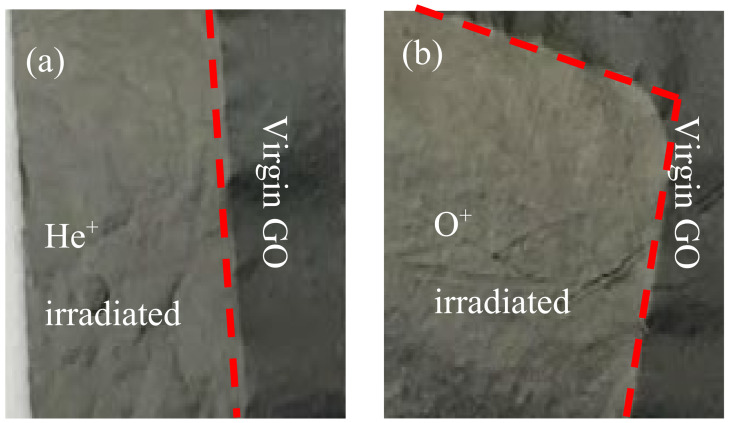
Magnified 5× optical image of the interface between virgin GO and the irradiated one by He^+^ ions (**a**) at the 5 × 10^13^ ions/cm^2^ fluence and by O^+^ ions (**b**) at the 5 × 10^12^ ions/cm^2^ fluence.

Morphological modifications on GO surfaces are shown in Figure 1 where the areas of GO foils irradiated by He^+^ ions (a) and O^+^ ions (b) at the 5 × 10^13^ ions/cm^2^ and 5 × 10^12^ ions/cm^2^ fluences, respectively, are separated by the red dotted mark from the virgin ones.

It is possible to note how the black color in the virgin GO turns into a metallic grey color after the He^+^ and O^+^ ions irradiations. The surface of the virgin GO appears more compact and smoother compared to that of the GO foil irradiated by He^+^ (see Figure 1a) and by O^+^ (see Figure 1b).

### 2.1. Rutherford Backscattering Spectrometry and Elastic Recoil Detection Analyses

The evaluation of the compositional changes in GO under the ion irradiation of H^+^, He^+^, O^+^ at low fluences are summarized in Table 1. The RBS/ERDA percentage atomic concentration of GO foils is reported before and after irradiation using ions with a 3 MeV energy and fluences ranging between 5 × 10^11^ ions/cm^2^ and 5 × 10^14^ ions/cm^2^.

The C/O ratio increases from 1.66 in the virgin GO to 1.83 at the H^+^ fluence of 5 × 10^11^ ions/cm^2^, to 1.96 at the fluence of 5 × 10^13^ ions/cm^2^ for H^+^; to 1.80 at the He^+^ fluence of 5 × 10^11^ ions/cm^2^, to 2.32 at the fluence of 5 × 10^14^ ions/cm^2^ for He^+^; to 1.85 at the O^+^ fluence of 5 × 10^11^ ions/cm^2^, to 2.62 at the fluence of 5 × 10^13^ ions/cm^2^ for O^+^ indicating a correspondence between the increase of released oxygen and the increase of the atomic number of the irradiating ions.

**Figure 2:**
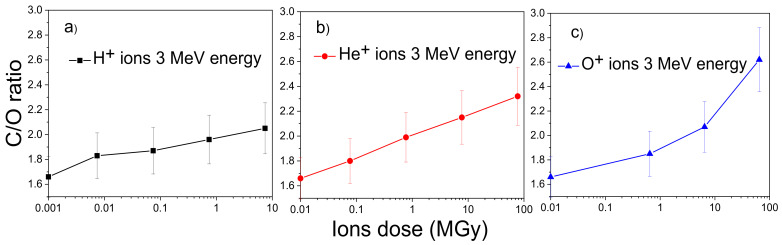
C/O ratio vs. doses released by H^+^ (**a**), He^+^ (**b**) and O^+^ ions (**c**).

The increase of the total stopping power (see Table 4) from 15.72 keV/μm to 164.22 keV/μm to 1374.70 keV/μm results in a major local energy deposition with consequent greater release of oxygen content from the GO foil. Figure 2 displays the C/O ratios obtained by RBS as a function of the doses released on the GO foils by different types of ions at the same experimental conditions.

In Figure 2a, the value for the C/O ratio at about 10 MGy is considered for a better comparison with those obtained at higher doses released by the He^+^ and O^+^ ions.

The error in the evaluation of C/O ratio is about 10%. An almost linear relation between the RBS C/O ratio and the absorbed dose is visible, within the experimental error, for all three beams at lower doses than 750 kGy and in the regime of electronic energy deposition. For higher doses the linearity decreases, and a saturation trend appears proportionally to the regime of nuclear energy deposition.

Therefore, Figure 2 allows us to assert that when the electronic stopping power prevails (for protons and helium beams), there is a good linearity between C/O ratio and the ion dose up to about 100 MGy, while when the nuclear stopping power is no longer negligible (for oxygen beams) the linearity occurs only at low dose because at high dose the effects produced by the ion collisions and the higher energy deposition enhance the C/O ratio over the linearity. These data confirm that GO is a good radiation detector and it could be a promising dosimeter as reported in the literature [19,20,21].

The H^+^ and He^+^ ion beams pass through the GO foil having a greater projected range than the GO thickness. In this case, the electronic energy loss dominates on the nuclear one as one can see in Table 4; it is responsible for both ionizations and partial chemical bond ruptures inducing the GO deoxygenation as the fluence increases. The presence of Mn, S and N is attributable to the synthesis method of GO.

When the 3 MeV oxygen ions hit the GO foil, they lose kinetic energy due to the elastic and inelastic collisions in the irradiated material, but as the GO foil is thicker than their projected range they stop inside the foil implanting themselves. Owing to the predominance of the electronic stopping power, the atomic ionization and the electron excitation result in both new highly reactive radicals and cross-linking effects. The nuclear stopping power is yet small even it is increasing on going from H^+^, to He^+^, to O^+^ as one can see in Table 4.

### 2.2. Scanning Electron Microscopy Analysis

**Figure 3:**
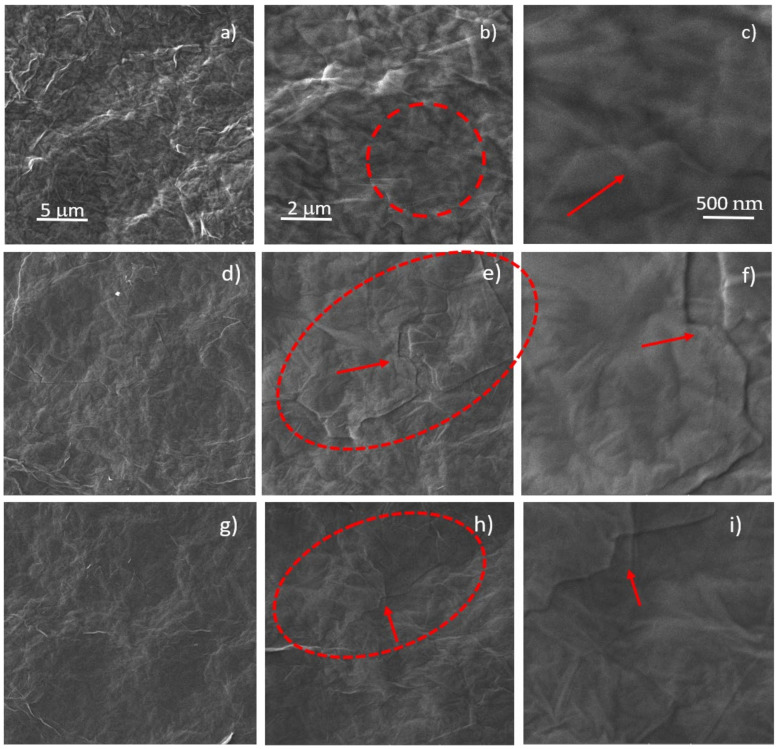
SEM images of GO foils irradiated by the 3 MeV H^+^ ions at the 5 × 10^13^ ions/cm^2^ (**a**–**c**), He^+^ ions at 5 × 10^12^ ions/cm^2^ (**d**–**f**) and O^+^ ions at 5 × 10^12^ ions/cm^2^ (**g**–**i**) fluences (A colour version of this figure can be viewed online).

The morphological study has been performed by SEM. Images with magnifications of 6.92 kX, 20.8 kX, 69.2 kX for view fields of 30 μm, 10 μm, 3 μm respectively and at the H^+^ 5 × 10^11^ ions/cm^2^ (a–c), 5 × 10^12^ ions/cm^2^ (d–f), 5 × 10^13^ ions/cm^2^ (g–i) fluences are shown in Figure 3. Further SEM images of the virgin and ion irradiated GO are presented in Figures S1–S3 of the Supplementary Materials.

In Figure 3b,e,h, the dashed red circles indicate the areas of interest; in Figure 3c,f,i the red arrows reveal the same corresponding areas of Figure 3b,e,h but magnified at 200 kX.

Figure 3b displays the surface of GO irradiated by H^+^ ions at the 5 × 10^13^ ions/cm^2^ fluence and the marked area by red dashes consists of lifted graphene flakes. In Figure 3c the red arrow indicates the magnified red marked area of GO foil of Figure 3b, suggesting that the lifting of graphene sheets may be caused by the outgassing of the oxygen functional groups and water removed by the ion irradiation process with H^+^ ions at the 5 × 10^13^ ions/cm^2^ fluence. The same effect is induced on the He^+^ and O^+^ irradiated GO is clearly visible at 5 × 10^12^ ions/cm^2^ in Figure 3d–f and Figure 3g–i respectively.

### 2.3. Atomic Force Microscopy (AFM) Analysis

**Figure 4:**
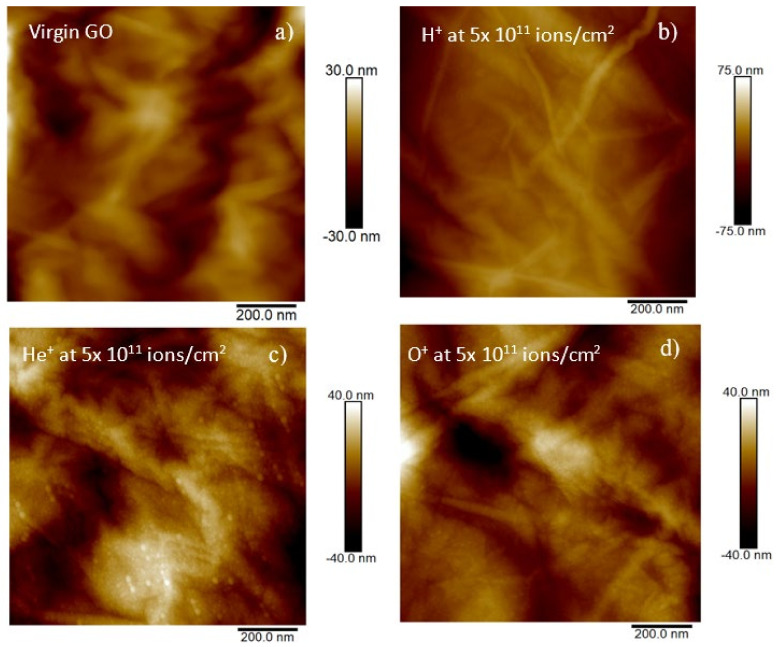
2D AFM images (scan area of 1 × 1 μm) in the virgin GO (**a**), GO irradiated by H^+^ at 5 × 10^11^ ions/cm^2^ (**b**), GO irradiated by He^+^ at 5 × 10^11^ ions/cm^2^ and (**c**), GO irradiated by H^+^ at 5 × 10^11^ ions/cm^2^ (**d**).

Further study of the morphological modification on GO foils ion irradiated has been carried out by atomic force microscopy. Figure 4 shows the AFM images at the same view field of 1 μm, obtained in the contact mode on virgin GO (see Figure 4a), and on GO irradiated by H^+^ (see Figure 4b), He^+^ (see Figure 4c) and O^+^ (see Figure 4d) ions at the same fluence of 5 × 10^11^ ions/cm^2^. The surface of virgin GO appears very rough and irregular with a maximal height of the profile on the order of tens of nm.

The maximal height of the virgin GO profile obtained by height cross-sectional profile is about 2.1 μm. In Table 2 are listed the values of the average roughness (Ra) and of the root mean square (RMS) for the virgin and the ion irradiated GO at the same fluence and same view field.

The Ra in virgin GO is 40.2% and 15.6% lower than in GO irradiated at the fluence of 5 × 10^11^ ions/cm^2^ by H^+^ and He^+^ respectively. The RMS in virgin GO is 43.0% and 20.4% lower than in GO irradiated at a fluence of 5 × 10^11^ ions/cm^2^ by H^+^ and He^+^, respectively.

This result agrees with the literature [28] according to which the roughness increases in the GO foils irradiated at the same dose in the regime of electronic energy loss. With respect to the O^+^ irradiation, an opposite trend is observed.

In fact, the Ra in virgin GO is 17.3% higher than in GO irradiated by O^+^ at the fluence of 5 × 10^11^ ions/cm^2^ and its RMS is 3.8% higher than in GO irradiated by O^+^ at the fluence of 5 × 10^11^ ions/cm^2^. This could be due to the sputtering effects of O^+^ ions on the GO surface.

**Figure 5:**
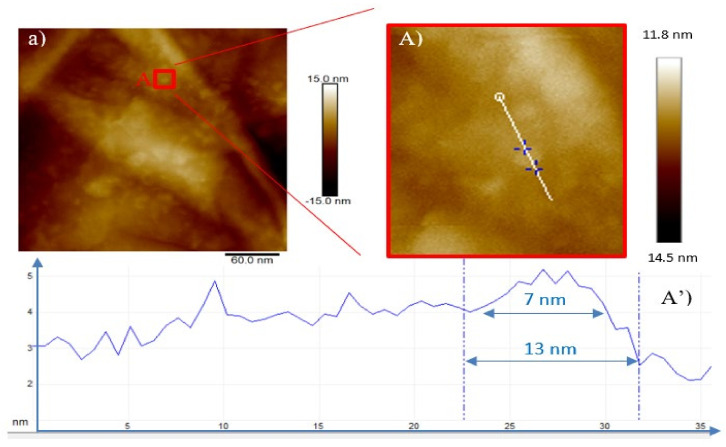

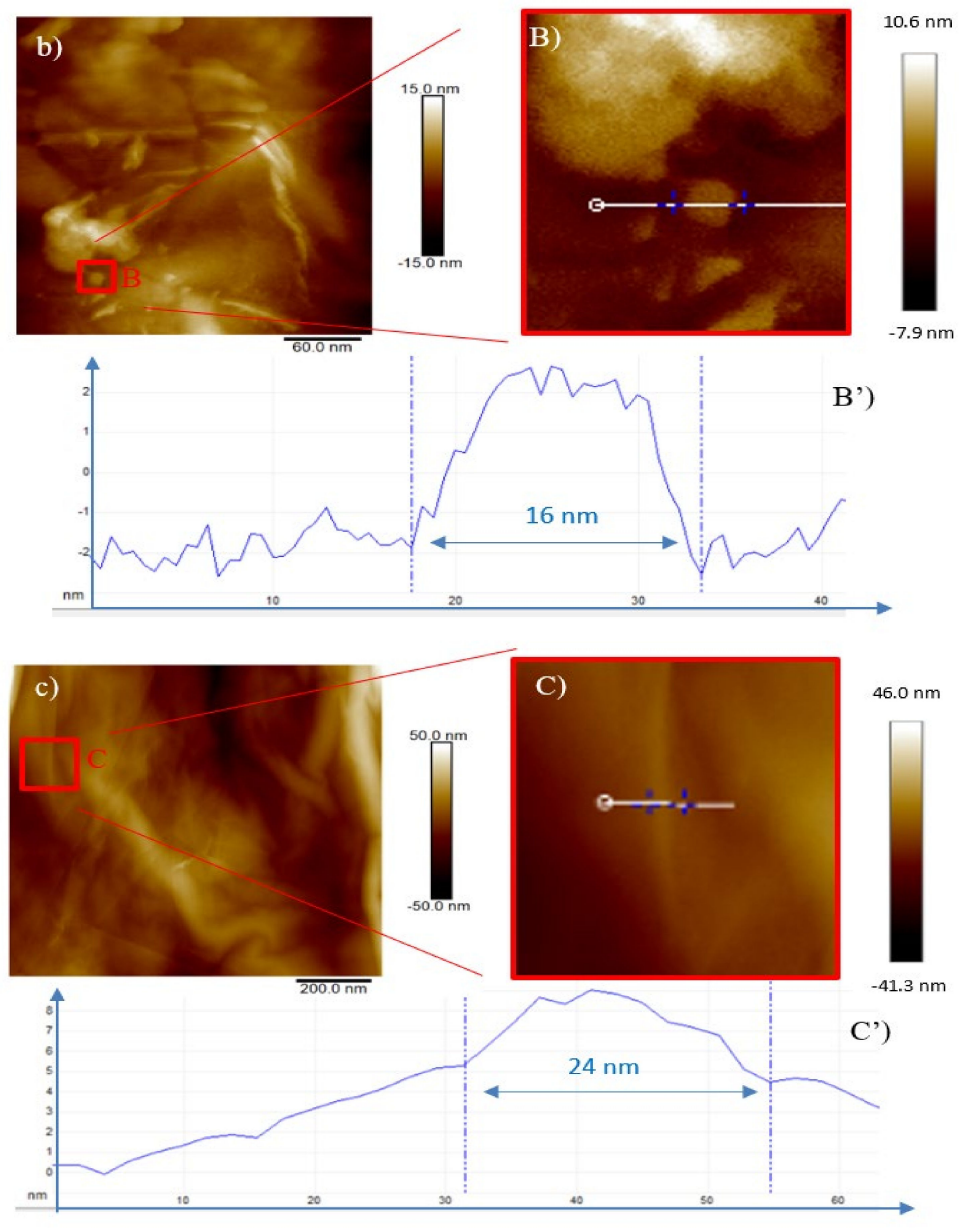
2D AFM images of GO irradiated by the 3 MeV H^+^ ions at the 5 × 10^11^ ions/cm^2^ (**a**), at the 5 × 10^12^ ions/cm^2^ (**b**) and at the 5 × 10^13^ ions/cm^2^ (**c**) fluences. Magnified areas of the red squares (**A**–**C**) and profiles of the corresponding selected structures (**A’**–**C’**), respectively.

**Figure 6:**
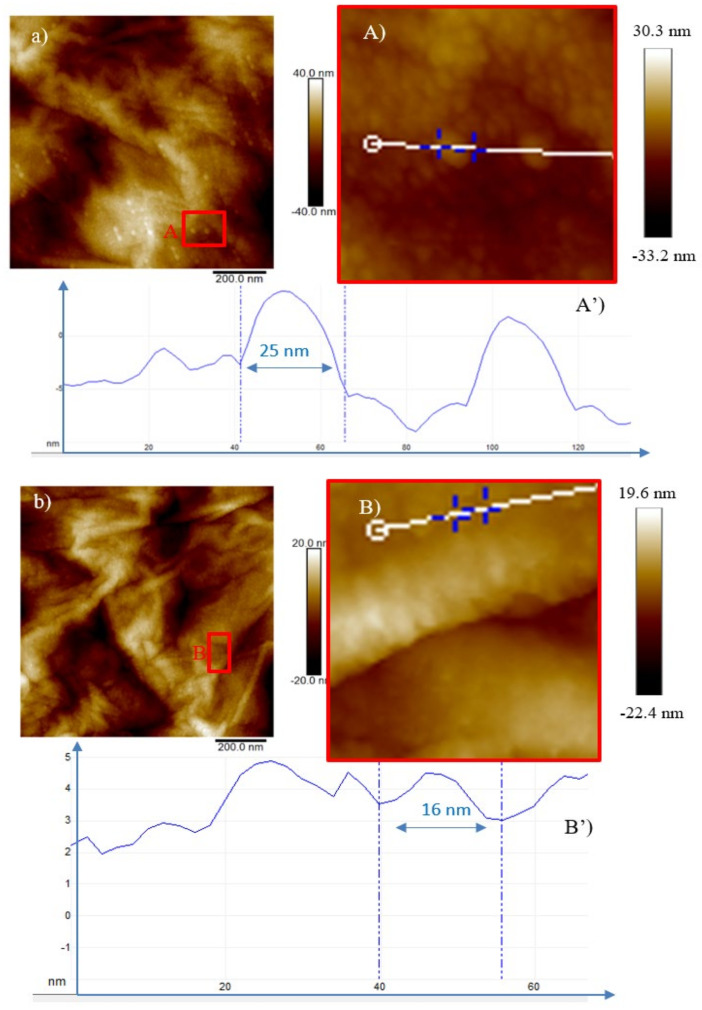
2D AFM images of GO irradiated by the 3 MeV H^+^ ions at the 5 × 10^11^ ions/cm^2^ fluence (**a**), magnified area of the red square (**A**) and profile of the selected structure (**A’**) and at the 5 × 10^12^ ions/cm^2^ fluence (**b**), magnified area of the red square (**B**) and profile of the selected structure (**B’**).

**Figure 7:**
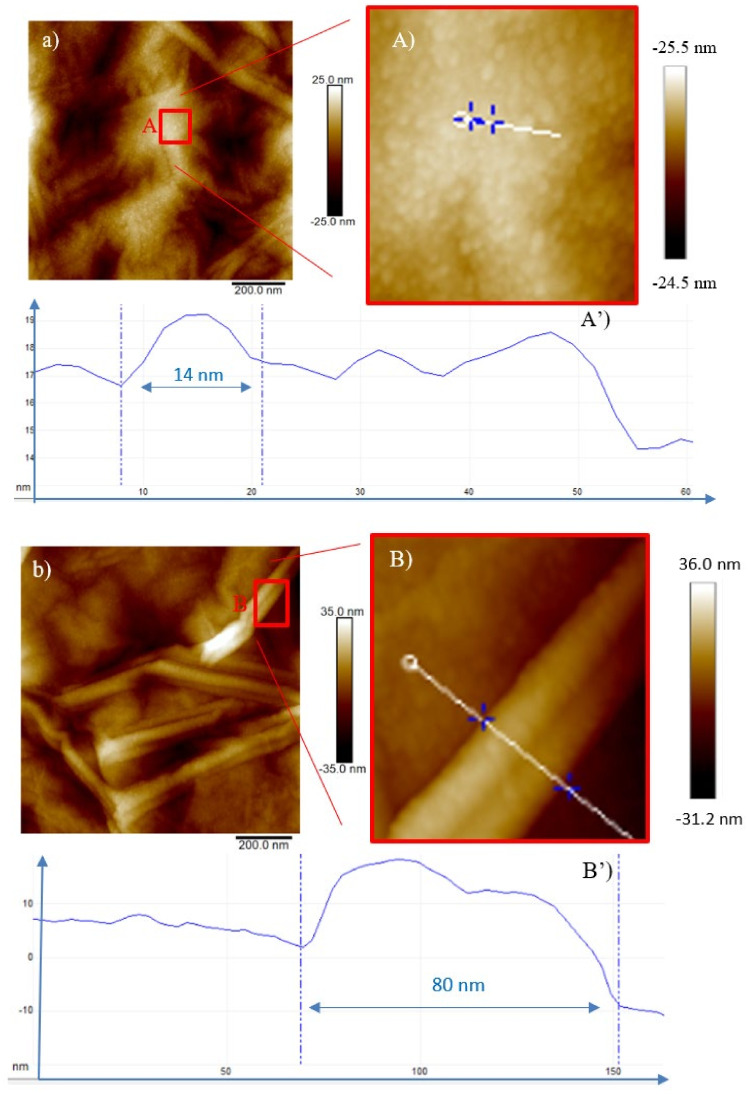
Two-dimensional AFM images of GO irradiated by the 3 MeV He^+^ ions at: the 5 × 10^13^ ions/cm^2^ fluence (**a**), magnified area of the red square (**A**) and profile of the selected structure (**A’**) and at the 5 × 10^14^ ions/cm^2^ fluence (**b**), magnified area of the red square (**B**) and profile of the selected structure (**B’**).

**Figure 8:**
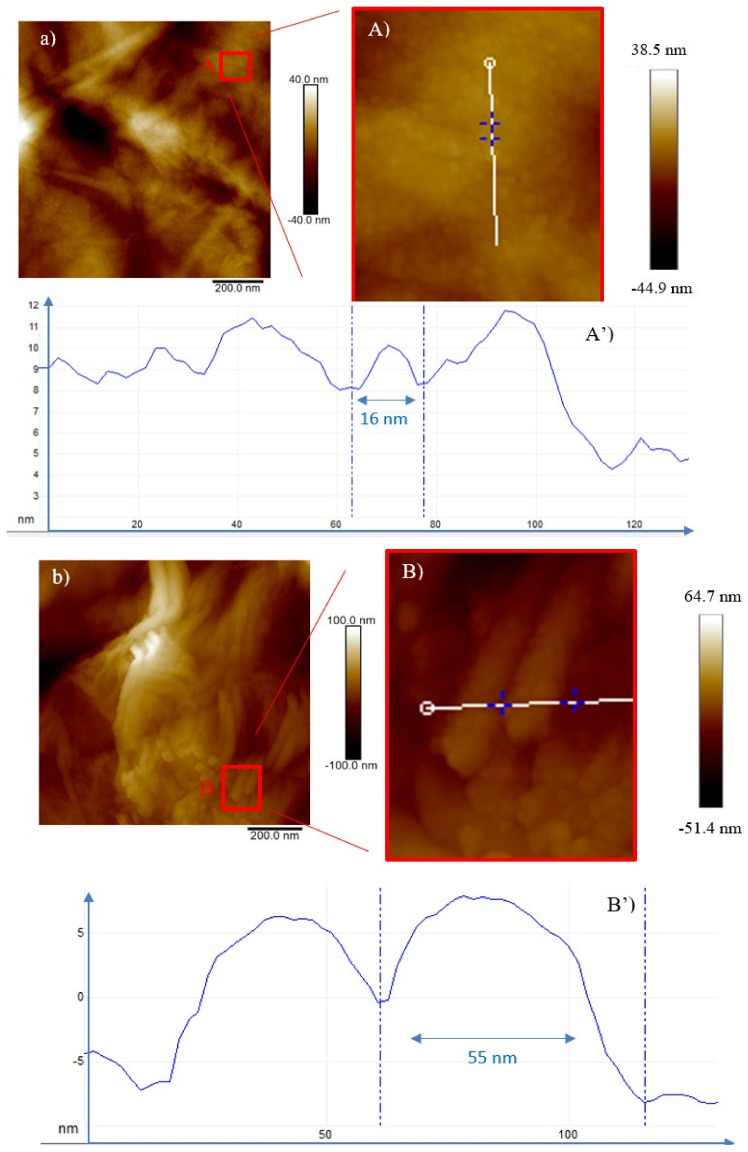
Two-dimensional AFM images of GO irradiated by the 3 MeV O^+^ ions at the 5 × 10^11^ ions/cm^2^ (**a**), magnified area of the red square (**A**) and profile of the selected structure (**A’**) and at the 5 × 10^12^ ions/cm^2^ fluence (**b**), magnified area of the red square (**B**) and profile of the selected structure (**B’**).

**Figure 9:**
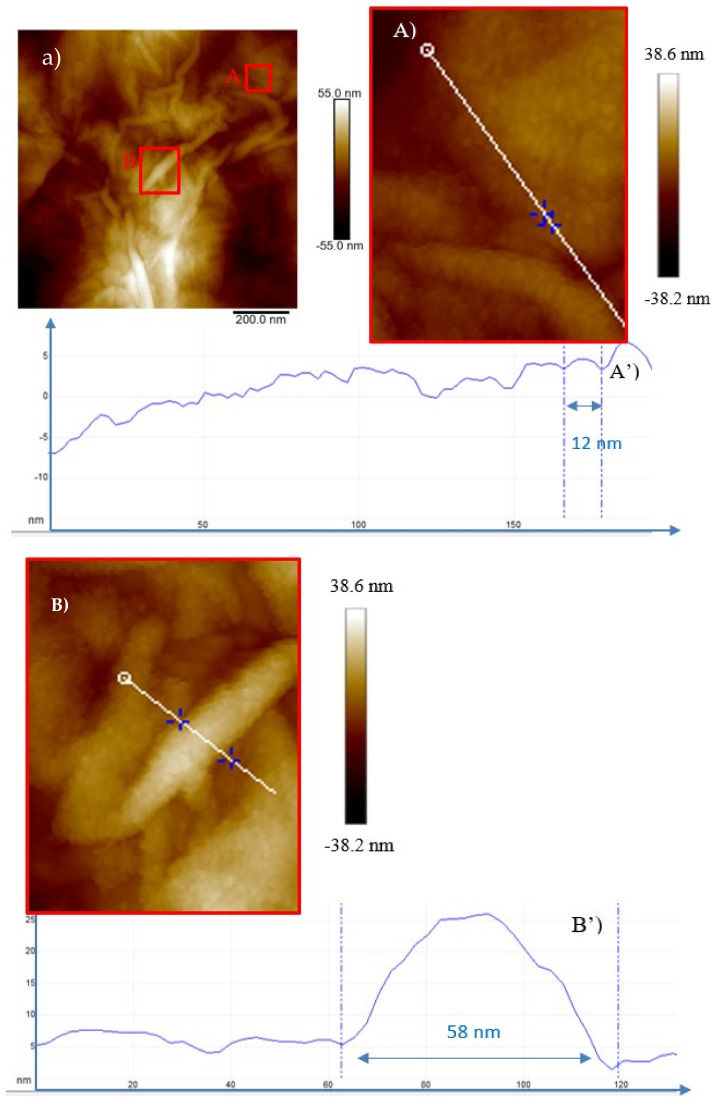
Two-dimensional AFM images of GO irradiated by the 3 MeV O^+^ ions at the 5 × 10^13^ ions/cm^2^ (**a**), magnified area of the red square (**A**) profile of the selected structure (**A’**) and magnified area of the red square (**B**) and profile of the selected structure (**B’**).

Figure 5, Figure 6, Figure 7, Figure 8 and Figure 9 show the AFM images, in 2 dimensions, obtained in the contact mode for the GO foils irradiated with H^+^, He^+^ and O^+^ ions at the investigated fluences.

To create a preliminary distinction between particles, their volume-to-surface ratio could help. Conventionally, particles for which the height/diameter ratio is greater than 0.3 are considered to be 3D structures [29] and those with less than 0.3 two-dimensional or planar structures. Therefore, the AFM observed structures fall in the second class. Particles with distinct x-y dimensions are considered to be polyhedral while those with similar x-y dimensions spherical.

When GO is irradiated by 3 MeV H^+^ ions at the lowest investigated fluence (see Figure 5a) nanometric irregular bulges are observed which raise and lift up as the fluence increases (see Figure 5b,c). This is due to the H^+^ irradiation-induced exfoliation and rupture of some GO sheets. The structure of interest is marked in the red square in Figure 5A.

The profile (see Figure 5A’) obtained by AFM, indicates ranges between 7 and 13 nm in width and 1 nm in height. In Figure 5b, together with ripples, bending and rolled up graphene layers, circular structures of 16 nm diameter and 0.6 nm height (see Figure 5B’) can be recognized as well as tubular structures 10 nm in width and 1.3 nm in height. In Figure 5c, the number of features is less evident even if the structure marked in the red square (see Figure 5C) displays a 24 nm width and a 0.7 nm height (see Figure 5C’).

Further AFM images of the ion irradiated GO are presented in Figures S4–S6 of the Supplementary Materials.

When GO is irradiated by 3 MeV He^+^, ions at the lowest investigated fluence bright spots (see Figure 6a,A) are visible with nanometer dimensions of about 25 nm at the base and of 3 nm in height (see Figure 6A’). In Figure 6b the base size of the dots marked in the red square is of about 16 nm and the height of 0.5 nm, indicating a decrease in the base size and an increase in number.

Two-dimensional AFM images of GO irradiated by the 3 MeV He^+^ ions at the 5 × 10^13^ ions/cm^2^ (a) and at 5 × 10^14^ ions/cm^2^ (b) fluences are displayed in Figure 7a,b, respectively.

Specifically, the presence of these nano-hillocks could be explained by the ability of energetic ions to release high energy along their track, in a very small volume which can be assumed cylindrical and surrounding the ion trajectory. It could suggest that the localized reduction of GO occurring along the ion tracks can generate nanosized hillocks (bright spots) at the surface which could be considered graphene quantum dots (GQDTs) embedded in a non-conducting matrix as confirmed by literature [22]. The density of these QDs increases with the fluence (see red squares in Figure 6b and Figure 7a,b) and at high doses, they start to overlap [22].

The generation of tubular structures (see Figure 7b) such as carbon nanotubes (CNTs) or GO/graphitic nanofibers nanohybrids (GOGNFs) visible on the surface of GO at the greatest investigated fluence could be explained considering that when increasing the deposited ion energy to a certain threshold value (depending on the material), the electron excitations can result in higher lattice heating and defect formation assisting the rolling up and bending of graphene layers.

In the red square of Figure 7a the base size of the dots still decreases up to about 14 nm and height of 0.8 nm, while, in Figure 7b, tubular frameworks 40 nm base sized and 11 nm in height mixed to circular structures with a diameter less than 14 nm are revealed and marked in the red square. This occurs because in the case of He^+^ irradiated GO, like for H^+^ ions, the beam passes through the GO foil having a thickness of 10 μm. This causes that the electronic energy loss dominates, inducing the increasing of the fluence, the deoxygenation of GO, in agreement with the RBS analysis, its amorphization and the generation of new carbon phases.

In the case of GO irradiated by O^+^ (see Figure 8 and Figure 9), the beam is stopped in the foil. The predominance of the electronic stopping power induces changes in the composition and in the structural properties of the O^+^ irradiated GO.

However, the increased contribution of the nuclear stopping power promotes the deoxygenation in the oxygen implanted GO as confirmed by the RBS analysis.

In Figure 8a, the bright spots (QDs), marked in the red square, 16 nm in diameter and 0.2 nm in height (see Figure 8A’) were generated during the irradiation of GO by the O^+^ ions at the minimum fluence of 5 × 10^11^ ions/cm^2^ and at fluence of 5 × 10^12^ ions/cm^2^ give rise to CNTs or GOGNFs 55 nm in diameter and 7 nm in height (see Figure 8B’) as will be confirmed by the XRD and TEM data.

The number density of these structures increases with the fluence (see Figure 8A and Figure 9A), and at the highest investigated dose (see Figure 9a) carbon nanofibers [30] are formed as will be suggested by the XRD and TEM data. In the profile of Figure 9A, the diameter of the circular feature (see Figure 9A’) is reduced to 12 nm and the height to 0.08 nm while the tubular structures in the red square (see Figure 9B) display a 58 nm base and a height of 2.5 nm in Figure 9B’.

AFM analysis indicates a decrease of the GQDTs sizes from 25 nm to 14 nm (He^+^), and from 15 nm to 10 nm (O^+^) with increasing ion fluence in agreement with the literature [26].

### 2.4. Transmission Electron Microscopy (TEM) Analysis

**Figure 10:**
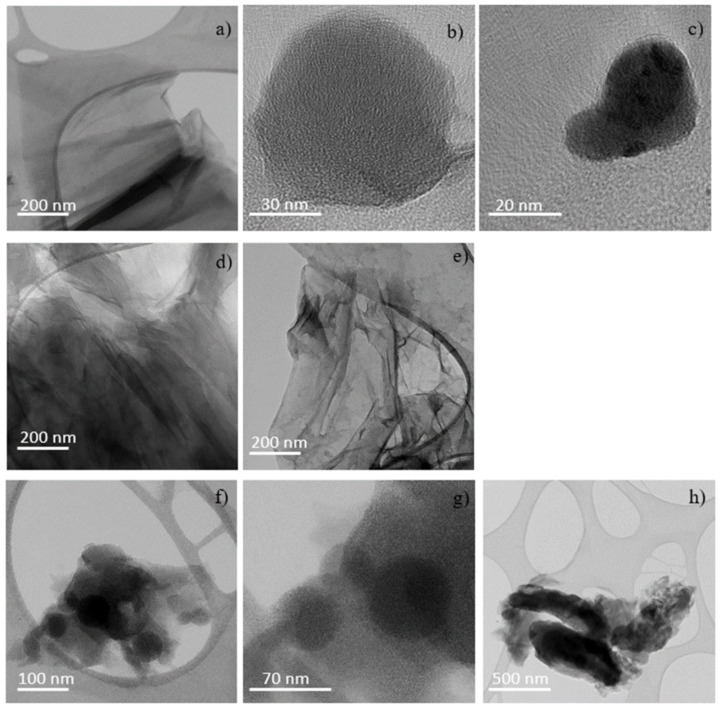
TEM micrograph of GO irradiated by the 3 MeV H^+^ ions at the 5 × 10^11^ ions/cm^2^ (**a**–**c**), 5 × 10^12^ ions/cm^2^ (**d**,**e**) and 5 × 10^13^ ions/cm^2^ (**f**–**h**) fluences.

To better define the structures deduced by AFM, TEM measurements have been carried out. TEM image related to the virgin GO presented in Figure S7 of the Supplementary Materials shows the presence of platelets and none of the structures noted on the GO ion have irradiated. Figure 10 shows TEM images obtained from the GO foils irradiated with H^+^. The progressive rippling effects pronounced with the absorbed fluence is visible in Figure 10a–h. In the first row from the top (see Figure 10a–c) the structures, formed in GO irradiated at the fluence of 5 × 10^11^ H^+^ ions/cm^2^, are shown; in the second row (see Figure 10d,e) GO is exposed at the fluence of 5 × 10^12^ H^+^ ions/cm^2^ and in the third row (see Figure 10f–h) at the fluence of 5 × 10^13^ H^+^ ions/cm^2^. Rippling and bending of graphene layers are displayed in Figure 10a–g together with almost circular structures, with sizes ranging between 15 nm and 60 nm, ascribable, in agreement with the literature [18] to graphene or GO quantum dots (GQDTs or GOQDTs). The 25 nm and 50 nm dots and the 70–250 nm wide and 1 mm long tubular structures are displayed in Figure 10h, attributable to GQDTs or GOQDTs and GOGNFs.

**Figure 11:**
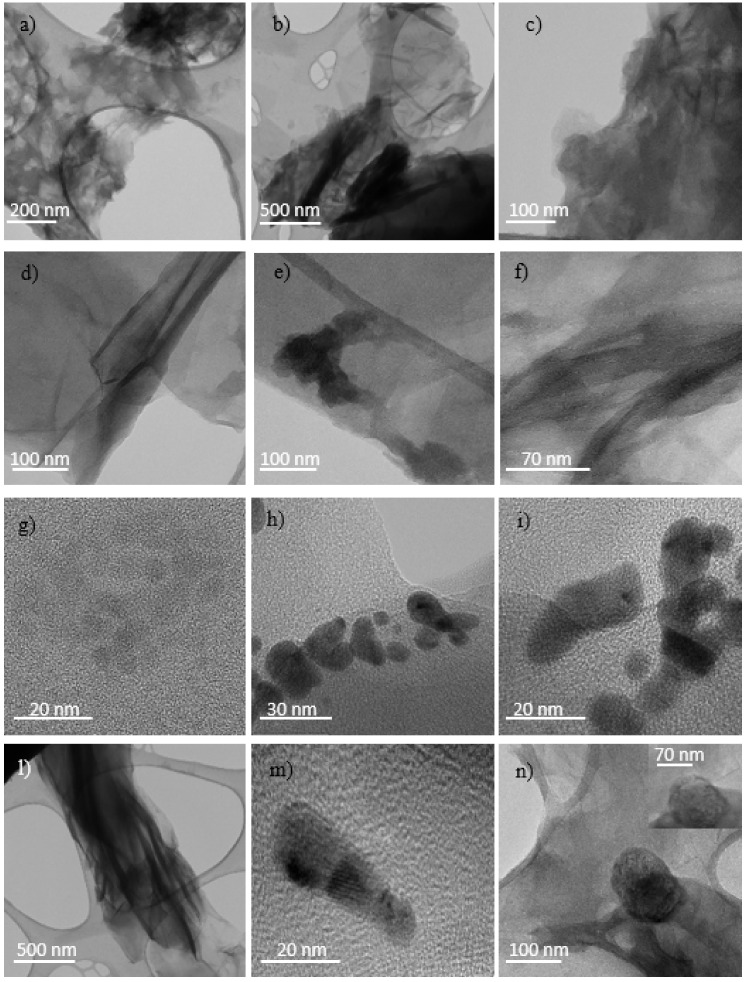
TEM micrograph of GO irradiated by the 3 MeV He^+^ ions at the 5 × 10^11^ ions/cm^2^ (**a**–**c**), 5 × 10^12^ ions/cm^2^ (**d**–**f**), 5 × 10^13^ ions/cm^2^ (**g**–**i**) and 5 × 10^14^ ions/cm^2^ (**l**–**n**) fluences.

In the first row from the top of Figure 11a–c, multilayer graphitic nanosheets (MGNs) unevenly distributed, are shown when GO is exposed to He^+^ at fluence of 5 × 10^11^ ions/cm^2^. In the second row (see Figure 11d–f), the fluence at the value of 5 × 10^12^ ions/cm^2^ is increased and some nanoribbons (15 nm wide and 1 mm long) are forming. In the third row (see Figure 11g–i), furtherly increasing the fluence at the value of 5 × 10^13^ ions/cm^2^ graphene or GO quantum dots (GQDTs or GOQDTs) are forming with diameter ranging between 10 and 20 nm linked to each other. In the last row (see Figure 11l–n) at the fluence of 5 × 10^14^ ions/cm^2^ tubular structures of different sizes, probably GOGNFs or CNTs deriving from the rolling up of the graphene sheets or the stacking of QDTs, and QDTs with diameter of 15–20 nm are visible.

This agrees with the literature [20] according to which the GO ion irradiation results in a progressive reduction of GO films. However, the loss of oxygen functional groups increases the structural instability of the reduced graphene oxide sheet, which tends to ripple more and more in proportion to the extent of the reduction and new structural phases form.

**Figure 12:**
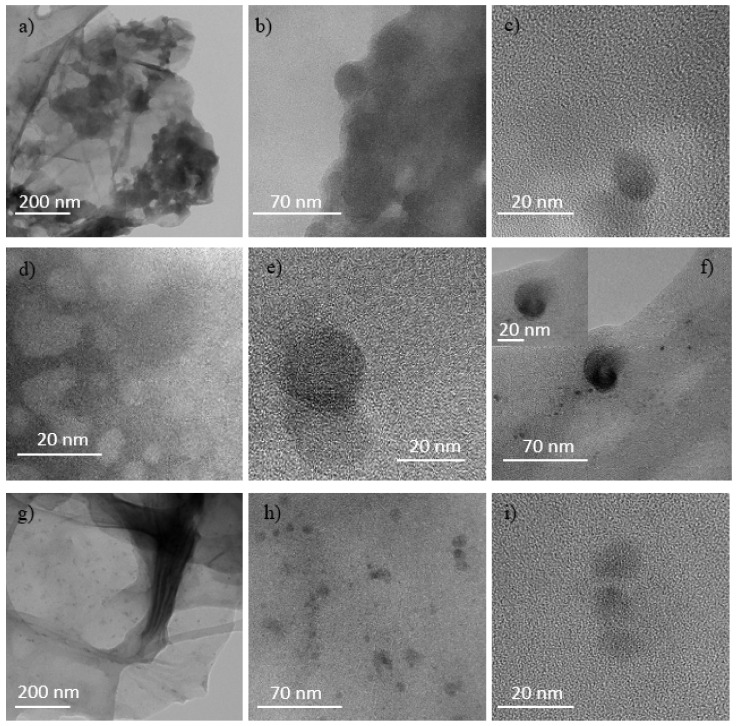
TEM micrograph of GO irradiated by the 3 MeV O^+^ ions at the 5 × 10^11^ ions/cm^2^ (**a**–**c**), 5 × 10^12^ ions/cm^2^ (**d**–**f**) and 5 × 10^13^ ions/cm^2^ (**g**–**i**) fluences.

In the first row from the top (see Figure 12a–c) the structures formed in GO implanted by O^+^ at the fluence of 5 × 10^11^ ions/cm^2^ are shown, in the second row (see Figure 12d–f) at the fluence of 5 × 10^12^ ions/cm^2^, in the third row (see Figure 12g–i) at the fluence of 5 × 10^13^ ions/cm^2^.

Multilayer graphitic nanosheets (MGNs) unevenly distributed and QDs with sizes of about 15 nm are displayed in Figure 12a–c and of about 10 nm in Figure 12h,i. In particular, in Figure 12e,f stacked-cup carbon nanofibers [30] with sizes less than 20 nm are shown. In Figure 12g some MGNs partially roll up giving rise to the tubular structures (probably CNTs or GOGNFs) observed in the AFM data. In the sample irradiated with O^+^ (see Figure 12), the total energy loss is higher due to the slightly increased contribution of the nuclear type and the ion-atom collisions producing collision cascades are responsible of the marked wrinkling of the rGO flakes and of the structural damage.

### 2.5. X-ray Diffraction Analysis

The changes in the GO structures after the irradiation of 3 MeV energy H^+^ ions (see Figure 10), He^+^ ions (see Figure 11) and O^+^ ions (see Figure 12) are also investigated as a function of both the ion atomic number and ion fluence by XRD to better identify the nature of the hypothesized QDTs. The open circles, displayed in all the XRD curves, indicate the experimental data, the continuous thick red lines are the best fits and the colored dashed lines represent the sub-bands. As one can see in Table 4, the H^+^ and He^+^ ions have a greater range than the GO foil thickness, while the O^+^ ions are significantly smaller. Accordingly, the H^+^ and He^+^ ions are not implanted into the GO foil and their energy deposition is mainly due to the electronic stopping power, while the O^+^ ions are implanted within the GO foils and their energy deposition besides the dominant electronic stopping power is affected by the growing contribution due to the nuclear stopping power (see Table 4). After the background subtraction, the experimental XRD curves were deconvoluted by using Gaussian–Lorentzian cross-product functions.

**Figure 13:**
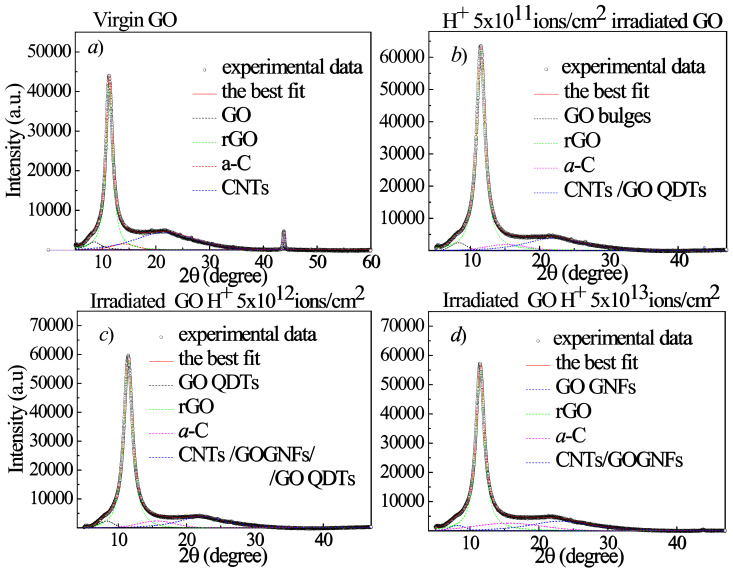
2θ/ω XRD curves of the virgin GO sample (**a**) and GO foils irradiated with 3 MeV H^+^ beams at increasing absorbed dose (**b**–**d**).

Figure 13a shows the GO pristine diffractogram consisting of a sharp peak at 11.3° preceded by a weak and broad shoulder at about 8.5° and followed by a very broad structure that is well fitted by three sub-bands centered at 14.0°, 21.7° and 43.8°.

The weak band at 8.5° and the strong diffraction peak at 11.3° corresponding to an interlayer spacing d of 1.04 nm and 0.78 nm, respectively, indicate that in the virgin GO foil there are areas containing different amounts of oxygen functional groups, due to the XRD measurement duration which induces some reduction. We label the weak band at 8.5°, with more content of oxygen functional group, as GO, while the 11.3° peak is labelled as rGO. As regards the features at about 14.0°, 21.7° and 43.8°, they are assigned, in agreement with the literature [16], to an amorphous carbon phase (a-C) and to carbon nanotubes (CNTs), these last ones due to the GO exfoliation with complete or partial rolling up of the graphene platelets. At the observed diffraction angle position CNTs can be present as single wall (SWCNTs) or multi wall (MWCNTs). When the GO foil is irradiated by the 3 MeV H^+^ ions at the lowest investigated fluence (see Figure 13b), the amount of rGO increases, thanks to an increased reduction process of GO, while the other XRD features decrease. On increasing fluence (see Figure 13c,d), the rGO diffraction peak decreases in intensity and in area due to its transformation in the other structural carbon phases, whose intensity and area increase as already observed in our previous paper for 2 MeV H^+^ ions [21]. In particular, in agreement with the literature [31,32,33], GO QDTs, CNTs and GOGFNs seem to contribute to the large XRD feature located between 22 and 23°.

**Figure 14:**
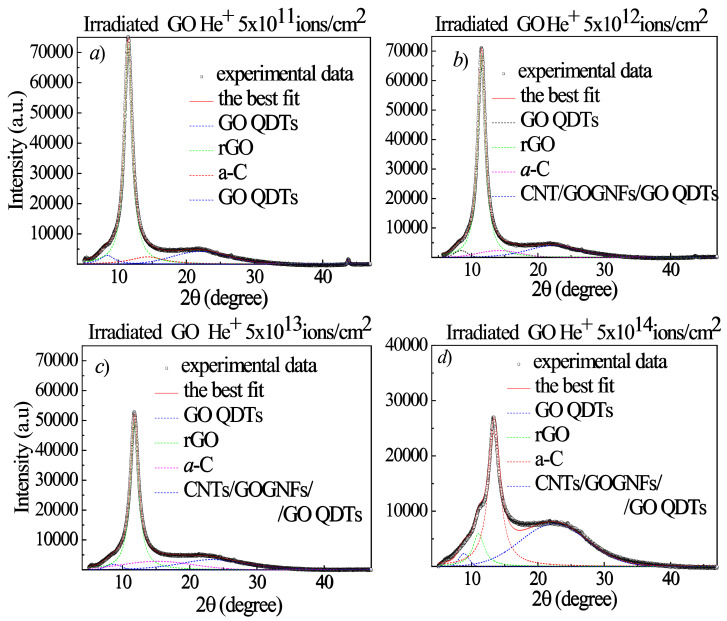
2θ/ω XRD curves of the GO foils irradiated with 3 MeV He^+^ beams at increasing absorbed dose (**a**–**d**).

In Figure 14a–d the XRD curves for the He^+^ irradiated GO are illustrated. When the GO foil is irradiated by the 3 MeV He^+^ ions up to the 5 × 10^12^ ion/cm^2^ fluence (see Figure 14a,b) the amount of rGO increases, thanks to an increased reduction process of GO, while the other XRD features decrease or, at the low fluences, give rise to GO QDTs as indicated by the AFM data and by the XRD features at about 8.4°, 22° and 43.8°, in agreement with the literature [31].

On increasing fluence (see Figure 14c,d), the rGO diffraction peak decreases in intensity and in area due to its transformation in the other structural carbon phases, whose intensity and area increase. Being the pristine GO platelets depauperated from some oxygen groups and closer allows them to turn themselves into amorphous carbon or to roll up, mainly forming CNTs or GOGNFs accompanied by GO QDTs (see Figure 14d), according to the above presented AFM data.

**Figure 15:**
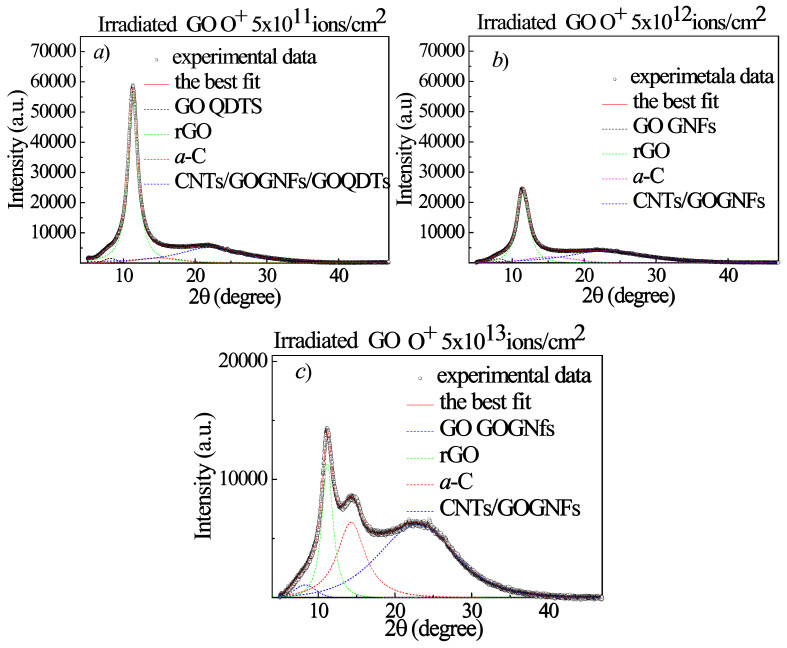
2θ/ω XRD curves of the GO foils irradiated with 3 MeV O ion beams at different absorbed doses (**a**–**c**).

In Figure 15a–c the XRD diffraction curves for the O^+^ implanted GO foils are illustrated.

When the GO foil is irradiated by the 3 MeV O^+^ ions at the lowest investigated fluence (see Figure 15a) the amount of rGO increases, thanks to an increased reduction process of GO, while the other XRD features decrease or give rise to GO QDs, CNTs or GOGNFs as indicated by the presence of the XRD features at about 8.2° and 22° in 2θ. The number density of these structures increases with the fluence (see Figure 15b–c). At the highest investigated fluence (see Figure 15c), in addition to decreasing the rGO diffraction peak in intensity and in areas in favor of the amorphous carbon, CNTs and GOGNFs, a slight increase of the GO peak area at about 8.3°, due to the oxygen implantation, is observed.

In Table 3, some XRD parameters deduced by the best fits such as the GO, a-C, rGO, GO QDTs. CNTs, GOGNFs intensity, position, FWHM and area are listed for GO irradiated by H^+^, He^+^, O^+^ ions at fluences ranging between 5 × 10^11^ ions/cm^2^ and 5 × 10^14^ ions/cm^2^.

The XRD curves reported in Figure 13 and Figure 14 suggest that for proton and helium ion beams the GO reduction occurs starting from low ion fluence inducing electronic energy loss. As a consequence of the ion irradiation, the GO reduction is the first process to occur followed by the a-C, GO bulges, GO QDTs, CNTs and GOGNFs formation. The ion irradiated GO foil is more compact than the virgin GO because the oxygen degassing is accompanied by a decrease in the interlayer distance making the platelets closer, by an increase of the amount of amorphous carbon, by a formation of GO QDTs or by the rolling up of the platelets, thus forming CNTs and GOGNFs.

The decreasing of the interlayer distance from 1.04 nm in the virgin GO to smaller values in the irradiated samples indicates narrower interlayer distances and more compact samples. The reduction of the interlayer distance up to values that are comparable with the graphite interlayer distance of about 0.332 nm, as a consequence of the ion energy deposition, supports the consideration that the irradiated GO foil exhibits higher density and more compactness.

According to the literature [22,35], at lower electronic stopping power Se values than about 7.2 keV/nm, at which a smaller heat release corresponds in the target material, at low fluences the structural damage is not significant, while at intermediate fluences localized rGO spots are generated. These rGO spots can be considered graphene QDTs embedded in a non-conducting matrix. At higher Se values than 9–18 keV/nm, as occurs in our case, the creation of defects can be responsible for the formation of sp-hybridized carbon chains which are bridges joining two sides of the nano-hole (i.e., antidot) created inside the ion track [22]. Therefore, ions with high Se values can form coupled QDTs-antidots with sp-C bridges [22]. Based on these observations, the here treated ion irradiation of GO foils probably will not give rise to “classical” tracks, but rather it will lead to discontinuous randomly distributed amorphous regions in agreement with what happens in graphite [35]. More detailed analyses will be performed to better identify the hypothesized carbon phases.

**Figure 16:**
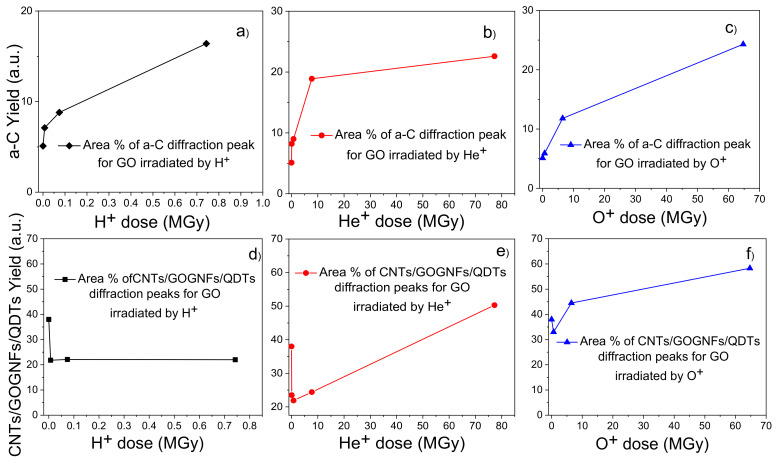
Areas of a-C (**a**–**c**) and CNTs/GOGNFs/GO QDTs (**d**–**f**) diffraction peaks vs. the ion absorbed dose for the GO foils irradiated by H^+^ (**a**,**d**), He^+^ (**b**,**e**) and O^+^ (**c**,**f**) ions.

The response to the dose absorbed from the GO foils irradiated by H^+^, He^+^ and O^+^ ions is shown in Figure 16 in terms of the yield of X-ray diffraction peaks attributable to the a-C and CNTs/GOGNFs/GOQDTs carbon structures as a function of ion dose. The values of the yields of a-C and CNTs/GOGNFs/GOQDTs are obtained from the areas of the relative diffraction peaks reported in Table 3.

The increase of the yield in a-C with the increase of the atomic number of the irradiating ions (see Figure 16a–c) suggests that the carbon amorphization increases in the high defect density regime. This increment is lower for the electronic energy loss regime and becomes higher for higher nuclear energy loss contribution, generating a higher amorphous phase through ion collision effects. The decrease of the CNTs/GOGNFs/QDTs yield observed in Figure 16d,e is ascribable to the electron energy loss regime produced by the proton irradiation, while the increment deduced for the O ion beam at the high doses (see Figure 16f) is due to the increased nuclear energy loss, demonstrating that these carbon structures are generated by both the local high energy deposition and the atomic collisions occurring along the ion tracks.

## 3. Materials and Methods

### 3.1. GO Foil Preparation

The dispersion of GO in water at a 0.4 wt% concentration was poured on a flat surface, dried at room temperature for 24 h and peeled off from the substrate. The mass density of the obtained GO foil was assessed on a 1 cm × 1 cm square GO foil accurately weighted and after having calculated its area and measured its thickness by an optical microscope following the procedure described in ref. [36]. The thickness of GO foils was 10 µm and the mass density was (1.40 ± 0.06) g/cm^3^ with an error of 2%.

### 3.2. Ion Irradiation

The GO foils were assembled on a copper holder and placed at a vacuum condition of about 10^6^ mbar in the implantation chamber of the ATRI MTF STU ion-beam laboratory [37] using the 6 MV Tandetron accelerator. The sample holder was maintained at room temperature and the beam incidence was at 7° with respect to the sample surface normal. The ions were produced by a HVEE 860 cesium sputter ion source or a NEC (National Electrostatics Corp.-Middleton, USA) Toroidal Volume Ion Source (TORVIS), depending on both the ion source availability and desired fluence. The ion beam was scanned across the sample surface. Then, 3 MeV singly charged protons, helium and oxygen ions were employed to irradiate the GO foils. The ion current density was maintained constant at about 0.3 μA/cm^2^, the beam spot was collimated on the sample to a 1 cm × 1 cm surface and the irradiation time varied from 25 s to 4 h.

The electronic (*S_e_*), nuclear (*S_n_*) and total (*S_t_*) stopping powers and the ion range^®^ were evaluated using the SRIM code [38], taking into account the nominal composition and density of GO. The calculation of the absorbed doses (*D_a_*) in GO was obtained assuming an average mass density of 1.7 g/cm^3^ for rGO congruently with our previous papers [19,20]. From the following equation:

*D_a_* (*Gy*) = *F_i_* · *S_t_* · (*1*/*z* · *ρ*) (1)

where *F_i_* is the ion fluence in ions/cm^2^ units, *S_t_* is the total stopping power given in keV/μm, *z* (Coulombs) is the charge state of the incident ion beam and *ρ* (g/cm^3^) is the mass density of the irradiated GO (i.e., *ρ*_rGO_), the absorbed dose Da was quantified in Gy units as reported in Table 4.

### 3.3. Rutherford Back Scattering (RBS) Spectrometry and Elastic Recoil Detection Analysis (ERDA)

The elemental composition of GO before and after the ion irradiation has been evaluated by the RBS and ERDA techniques using a beam of 2.5 MeV H^+^ ions at an incidence angle of 0°. The scattered proton particles have been monitored by an Ultra-Ortec PIPS silicon detector located at the scattering angle of 160°. The recoiled hydrogen atoms have been collected at a scattering angle of 35° by a silicon detector covered with a 12 μm mylar filter to stop the forward scattered proton ions and the heaviest nuclei. The ion current has been maintained at about 2.5 nA.

The energy scale spectra for RBS and ERDA have been collected every 500 s and then converted in the concentration depth profile using the SIMNRA simulation code [39]. RBS analysis has been employed to evaluate the C/O atomic ratio, while ERDA was used for the C/H atomic ratio.

### 3.4. Structure Analysis by XRD

The structure modifications of the pristine and ion irradiated GO foils have been studied by X-ray diffraction (XRD). A diffractometer equipped with a Cu Kα source (λ = 0.154 nm), produced by a PaNalytical 9 kW rotating-anode generator and operating at 45 kV and 200 mA has been used to acquire the X-ray diffraction curves. A primary parabolic multi-layered mirror partially suppresses the Cu Kβ line and a Ni filter, on the secondary side, and completely removes the β line. The virgin and the ion irradiated GO foils have been located onto a polished diffractionless Si plate which was cut in such a way that it did not diffract in the symmetric Bragg-Brentano geometry. Symmetric 2θ/ω XRD curves have been collected in the (5–60)° region in 2θ. Each XRD curve has been fitted using the Peakfit software based on a nonlinear least-square fitting method and Gaussian–Lorentzian cross-product functions as line shapes.

### 3.5. Scanning Electron (SEM) and Atomic Force (AFM) and Transmission Electron (TEM) Microscopies

The morphology, the micro-structure and the roughness of the virgin and deposited samples have been monitored by scanning electron (SEM) and atomic force (AFM) microscopies. A LYRA3 GMU system (TESCAN, Brno, Czech Republic) was employed in the secondary-electron mode at an acceleration voltage of 5 kV for the SEM analysis on areas of 30 × 30 μm, 10 × 10 μm and 3 × 3 μm. A Dimension ICON AFM apparatus (Bruker Corp., Bremen, Germany), operating in the PeakForce QNM (Quantitative Nanoscale Mechanical) imaging mode in air and equipped with a silicon Tip on a Nitride cantilever (SCANASYST-AIR Probe) with a spring constant of 0.4 N/m was used for the AFM analysis on areas of about 300 nm^2^ and 1 μm^2^. The data processing and the characterization of the formed carbon-based structures with corresponding height cross-sectional profile were performed with the NanoScope Analysis 1.80 32 bit software.

The TEM micrographs of the specimens have been obtained using a Jeol 2200 FS (Jeol, Akishima, Japan) microscope equipped with a TVIPS camera.

## 4. Conclusions

This study reports on the effects induced on the GO foil irradiated by ions at low fluences: new structural phases, such as rGO, a-C, GO QDTs, GOGNFs, CNTs, MGNs and stacked-cup carbon nanofibers, are produced by the reduction of the oxygen functional groups bonded to the graphene platelets.

Due to the reduction, the GO sheets approach increases the mass density of the material.

The reduction level has been monitored by the RBS analysis and it has been noted as being proportional to the absorbed dose, validating the use of GO foils as dosimeters for fluences ranged between 5 × 10^11^ and 5 × 10^13^ ions/cm^2^.

The structural modifications of the GO foils irradiated by the 3 MeV H^+^, He^+^ and O^+^ ions have been examined by the XRD technique.

The Bragg law has been used to evaluate the decrease of the average distance between the carbon planes influencing the increment of the material density from about 1.4 g/cm^3^ in virgin GO up to about 2.0 g/cm^3^, a value increasing with the dose towards the typical graphite value of 2.23 g/cm^3^.

RBS and ERDA have indicated the increase in the oxygen and the hydrogen reduction with both the dose and the atomic number of ions bombarding the GO foils.

The XRD curves show, for low LET radiations (the H^+^ and He^+^ ions with high *S_e_* and negligible *S_n_*), the formation of rGO that increases at low fluence but decreases at higher fluences to favor the generation of a-C phase, GO QDTs, GOGNFs, MGNs and CNTs, which increases with the ion dose. For high LET radiations (the O^+^ ions with higher *S_e_* and *S_n_*) the GO QDTs are already formed at low fluence and the higher lattice heating can be responsible for defect formation assisting the rolling up and bending of graphene layers and giving rise to GOGNFs and CNTs up to 5 × 10^12^ ions/cm^2^. At the highest investigated O^+^ ions fluence (5 × 10^14^ ions/cm^2^), in addition to a decrease of the rGO diffraction peak in intensity and in an area in favor of the a-C, CNTs and GOGNFs phases, a slight increase on the GO peak area, due to the oxygen implantation, is observed.

The results of the present study are a continuation of our previous works concerning the use of GO as a radiation detector of energetic ions and indicate a correlation between the C/O ratio, measured by RBS, and the absorbed dose. Even if the dosimeter linearity occurs up to 750 kGy, the calibration curve the dosimeter can be employed up to the dose of about 100 MGy.

The previous works demonstrated that at greater doses than 150 MGy the rGO content decreased and the GO foil underwent significant damage and structural changes. The advantages of such dosimeters would consist in their water equivalence, high biocompatibility and low millimetric size which can be read using XRD techniques offering high 3D spatial resolution. Furthermore, the irradiation parameters (ion type, fluence and ion energy) could be tailored to minimize the induced structural disorder or to increase the amorphization degree, and at high Se values to form coupled GO QDTs-antidots with sp-C bridges, CNTs and GOGNFs. Moreover, the presented measurements demonstrate that the GO reduction can be well controlled by the ion irradiation, allowing the tailoring of different rGO parameters such as density, amorphous carbon phase, GO QDTs, GOGNFs, CNTs and gas permeability, according to recent literature.

## Figures and Tables

**Table 1:** Elemental composition of virgin and ion irradiated GO foils at different fluences have been obtained by RBS/ERD analyses.

Sample		Ions 3 MeV Energy	Fluence (Ions/cm^2^)	Atomic (%)						
				C	O	H	Mn	S	N	C/O
**GO**	**VIRGIN**	0	0	41.6	25	30	0.2	0.7	2.5	1.66
	**#1**	H^+^	5 × 10^11^	44.2	24.2	29.0	0.1	0.5	2.0	1.83
	**#2**		5 × 10^12^	44.6	23.9	28.9	0.1	0.5	2.0	1.87
	**#3**		5 × 10^13^	46.1	23.5	28.3	0.1	0.5	1.5	1.96
**GO**	**#4**	He^+^	5 × 10^11^	44.3	24.6	28.5	0.1	0.5	2.0	1.80
	**#5**		5 × 10^12^	46.5	23.4	28.0	0.1	0.5	1.5	1.99
	**#6**		5 × 10^13^	48.4	22.5	27.5	0.1	0.5	1.0	2.15
	**#7**		5 × 10^14^	49.9	21.5	27.0	0.1	0.5	1.0	2.32
**GO**	**#8**	O^+^	5 × 10^11^	44.4	24.0	29.0	0.1	0.5	2.0	1.85
	**#9**		5 × 10^12^	46.8	22.6	28.0	0.1	0.5	2.0	2.07
	**#10**		5 × 10^13^	52.4	20.0	26.0	0.1	0.5	1.0	2.62

**Table 2:** List of Ra and RMS values as a function of the H^+^, He^+^ and O^+^ ion fluences.

Ions	Ions Fluence (Ions/cm^2^)	Ra (nm)	RMS (nm)
Unirradiated/virgin GO	None	9.2	10.5
H^+^	5 × 10^11^	15.4	18.4
He^+^	5 × 10^11^	10.9	13.2
O^+^	5 × 10^11^	7.6	10.1

**Table 3:** List of the intensity, position, FWHM and area of the main features detected in the XRD curves deduced by the best fits for the unirradiated and irradiated GO sample areas [34].

Sample		Intensity	Position 2θ (Degree)	FWHM (cm^−1^)	Area (%)
**GO virgin**	GO	2048.2	8.5	3.0	4.8
	rGO	41,843.4	11.3	1.5	52.1
	*a*-C	1500.0	14.0	5.5	5.1
	CNT	4426.8	21.7	13.0	36.7
	CNT	4660.4	43.8	0.3	1.3
	GO bulges	2429.1	8.3	2.6	1.7
**GO H^+^ 5 × 10^11^**	rGO	61,949.1	11.5	1.5	69.4
	*a*-C	1867.4	15.2	7.0	7.1
	CNTs/GO QDTs	3838.9	22.0	9.5	21.5
	CNTs/GO QDTs	1483.9	43.8	0.3	0.3
	GO QDTs	2323.8	8.3	2.4	1.7
**GO H^+^ 5 × 10^12^**	rGO	57,474.2	11.5	1.5	60.1
	*a*-C	2355.8	15.6	6.2	8.8
	CNTs/GOGNFs/QDTs	3881.7	22.27	7.5	20.4
	GOGNFs	1841.8	8.2	2.6	1.3
**GO H^+^ 5 × 10^13^**	rGO	54,513.7	11.5	1.5	61.6
	*a*-C	2597.6	15.6	11.7	16.4
	CNTs/GOGNFs	3280.9	23.0	10.2	20.7
	GO QDTs	2903.6	8.4	2.5	4.5
**GO He^+^ 5 × 10^11^**	rGO	72,869.5	11.5	1.4	68.3
	*a*-C	2400.0	14.3	6.0	8.2
	GO QDTs	4175.3	22.0	9.4	18.8
	GO QDTs	1540.5	43.8	0.3	0.2
	GO QDTs	2311.8	8.4	2.4	1.4
**GO He^+^ 5 × 10^12^**	rGO	69,000.8	11.5	1.4	69.1
	*a*-C	2337.4	14.2	7. 5	9.0
	CNTs/GOGNFs/GO QDTs	4080.9	22.2	9.0	20.5
	GO QDTs	1850.4	8.7	2.6	1.3
**GO He^+^ 5 × 10^13^**	rGO	49,382.9	11.8	1.5	56.7
	*a*-C	2837.4	15.2	12.3	18.9
	CNTs/GOGNFs/GO QDTs	3421.3	23.3	11.3	23.1
	GO QDTs	2307.7	8.8	1.8	1.1
**GO He^+^ 5 × 10^14^**	rGO	5934.1	11.1	2.1	9.3
	*a*-C	24,395.6	13.4	2.2	40.4
	CNTs/GOGNFs/GO QDTs	7676.9	22.4	11.8	49.2
	GO QDTs	1510.0	8.2	2.1	0.8
**GO O^+^ 5 × 10^11^**	rGO	56,471.2	11.3	1.6	61.0
	*a*-C	1700.0	15.0	7.3	5.9
	CNTs/GOGNFs/GO QDTs	5144.9	22.0	10.7	32.2
	GOGNFs	1309.5	8.1	2.2	1.1
**GO O^+^ 5 × 10^12^**	rGO	22,912.6	11.5	2.0	43.7
	*a*-C	1944.1	15.3	7.7	11.8
	CNTs/GOGNFs	3965.0	23.0	12.5	43.4
	GOGNFs	1058.0	8.3	3.3	2.5
**GO O^+^ 5 × 10^13^**	rGO	11,323.5	11.2	1.7	17.4
	*a*-C	6375.4	14.3	4.3	24.3
	CNTs/GOGNFs	6156.2	22.9	11.8	55.8

**Table 4:** List of experimental parameters adopted for the ion irradiation of GO foils in vacuum.

Parameters	Ions Irradiating the Go Foil									
	H^+^			He^+^				O^+^		
Energy (MeV)	3.0			3.0				3.0		
*S_e_* (keV/μm)	15.71			164.10				1369.00		
*S_n_* (keV/μm)	0.01			0.12				5.70		
*S_t_* = *S_e_* + *S_n_* (keV/μm)	15.72			164.22				1374.70		
Range *R* (μm)	113.4			14.5				3.7		
Fluence *F_i_* (5 × 10^11^/cm^2^)	1	10	100	1	10	100	1000	1	10	100
Dose *D_a_* (MGy)	0.0074	0.0743	0.7427	0.0770	0.7730	7.7280	77.2800	0.6460	6.4690	64.7000
Sample label	#1	#2	#3	#4	#5	#6	#7	#8	#9	#10

## Supplementary Materials

The following are available online at https://www.mdpi.com/article/10.3390/ijms232012563/s1.
