# Supplementary material for: Compositional and Structural Modifications by Ion Beam in Graphene Oxide for Radiation Detection Studies
Source: Int J Mol Sci. 2022 Oct 19;23(20):12563. doi: 10.3390/ijms232012563 (PMC9604086; doi:10.3390/ijms232012563)
Supplement: Supplementary file 1 [file ijms-23-12563-s001.zip › ijms-1927778-supplementary.pdf]

Figure S1 h) displays the surface of GO irradiated by H^+ ions at the $5x10^{13}$ ions/cm^2 fluence and the marked area by red dashes consists of lifted graphene flakes. In Figure 3 i) the red arrow indicates the red marked area of GO foil of Figure 3 h), magnified at 200 kX, suggesting that the lifting of graphene sheets may be caused by the outgasing of the oxygen functional groups and water removed by the ion irradiation process with H+ ions at the $5x10^{13}$ ions/cm^2 fluence.

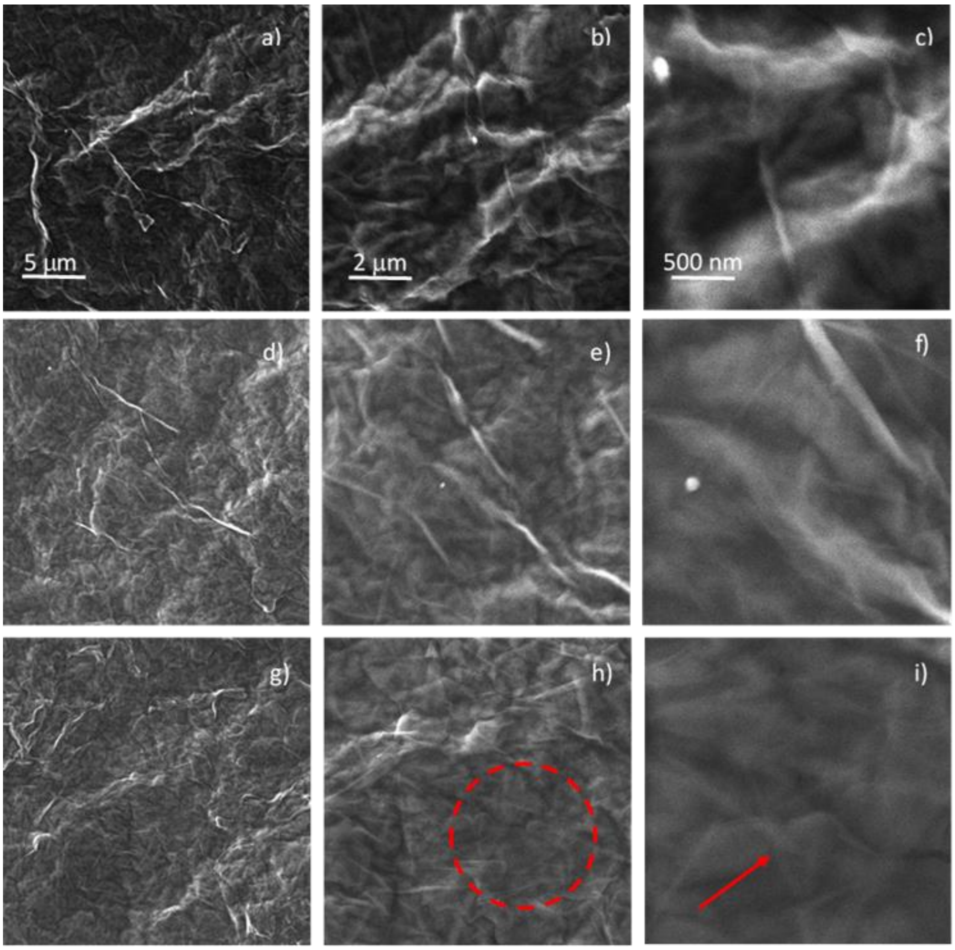

Figure S1. SEM images of GO foils irradiated by the 3 MeV H^+ ions at the $5x10^{11}$ ions/cm^2 a), b), c), $5x10^{12}$ ions/cm^2 d), e), f), $5x10^{13}$ ions/cm^2 fluences. In h) the dashed red circle indicates an area where the graphene sheets are raised; in i) the red arrow reveals the same area of h) but magnified at 200 kX. (A colour version of this figure can be viewed online).

Figure S2 shows the SEM images with magnifications of 6.92 kX, 20.8 kX, 69.2 kX for view fields of 30 μm, 10 μm, 3 μm respectively and at the He^+ 5x10^{11} ions/cm^2 a), b), c), 5x10^{12} ions/cm^2 d), e), f), 5x10^{13}ions/cm^2 g), h), i), 5x10^{14} ions/cm^2 l), m), n) fluences. The areas of the foils, where the lifted graphene flakes are distinctly visible, are marked using red dashes. This effect is induced on the He^+ irradiated GO already at 5x10^{12} ions/cm^2 which is the minimum applied ion fluence.

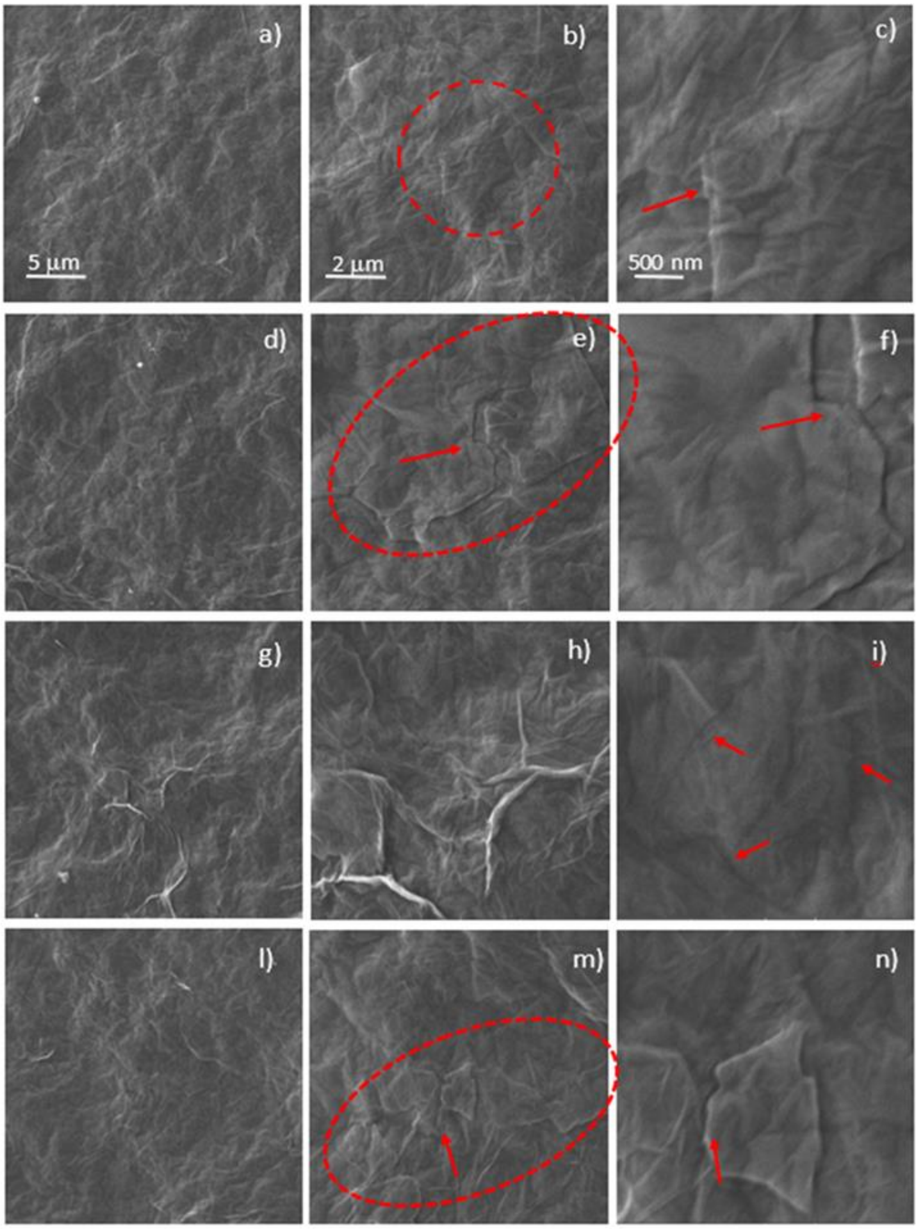

Figure S2. SEM images of GO foils irradiated by the 3 MeV He^+ ions at the 5x10^{11} ions/cm^2 a), b), c), 5x10^{12} ions/cm^2 d), e), f), 5x10^{13}ions/cm^2 g), h), i), 5x10^{14} ions/cm^2 l), m), n) fluences. The red dashed lines indicate GO areas in which the lifting of the graphene sheets is visible, while the red arrows the same magnified areas. (A colour version of this figure can be viewed online).

Figure S3 shows the O^+ SEM images with magnifications of 6.92 kX, 20.8 kX, 69.2 kX for view fields of 30 μm, 10 μm, 3 μm respectively and the 5x10^{11} ions/cm^2 a), b), c), 5x10^{12} ions/cm^2 d), e), f), 5x10^{13}ions/cm^2 g), h), i) fluences. The areas of the foils, where the graphene flakes are distinctly visible, are marked using red dashes. The red arrows indicate the magnified area where the density was modified by the O^+ ions at the fluence of 5x10^{14} ions/cm^2.

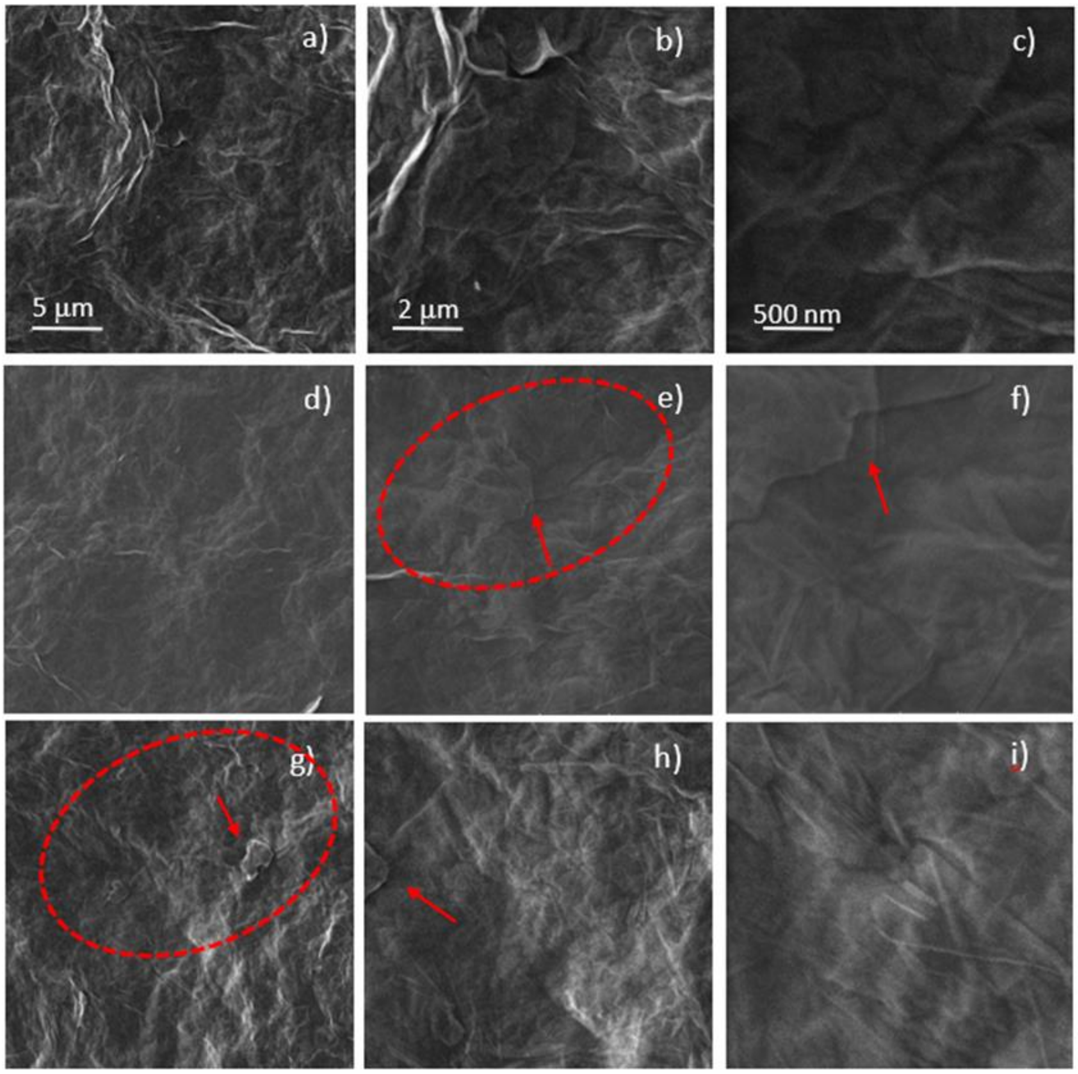

Figure S3. SEM images of GO foils irradiated by the 3 MeV O^+ ions at the 5x10^{11} ions/cm^2 a), b), c), 5x10^{12} ions/cm^2 d), e), f), 5x10^{13}ions/cm^2 fluences. The red dashed lines indicate GO areas in which the lifting of the graphene sheets is visible, while the red arrows the same magnified areas. (A colour version of this figure can be viewed online).

H^+ IRRADIATION

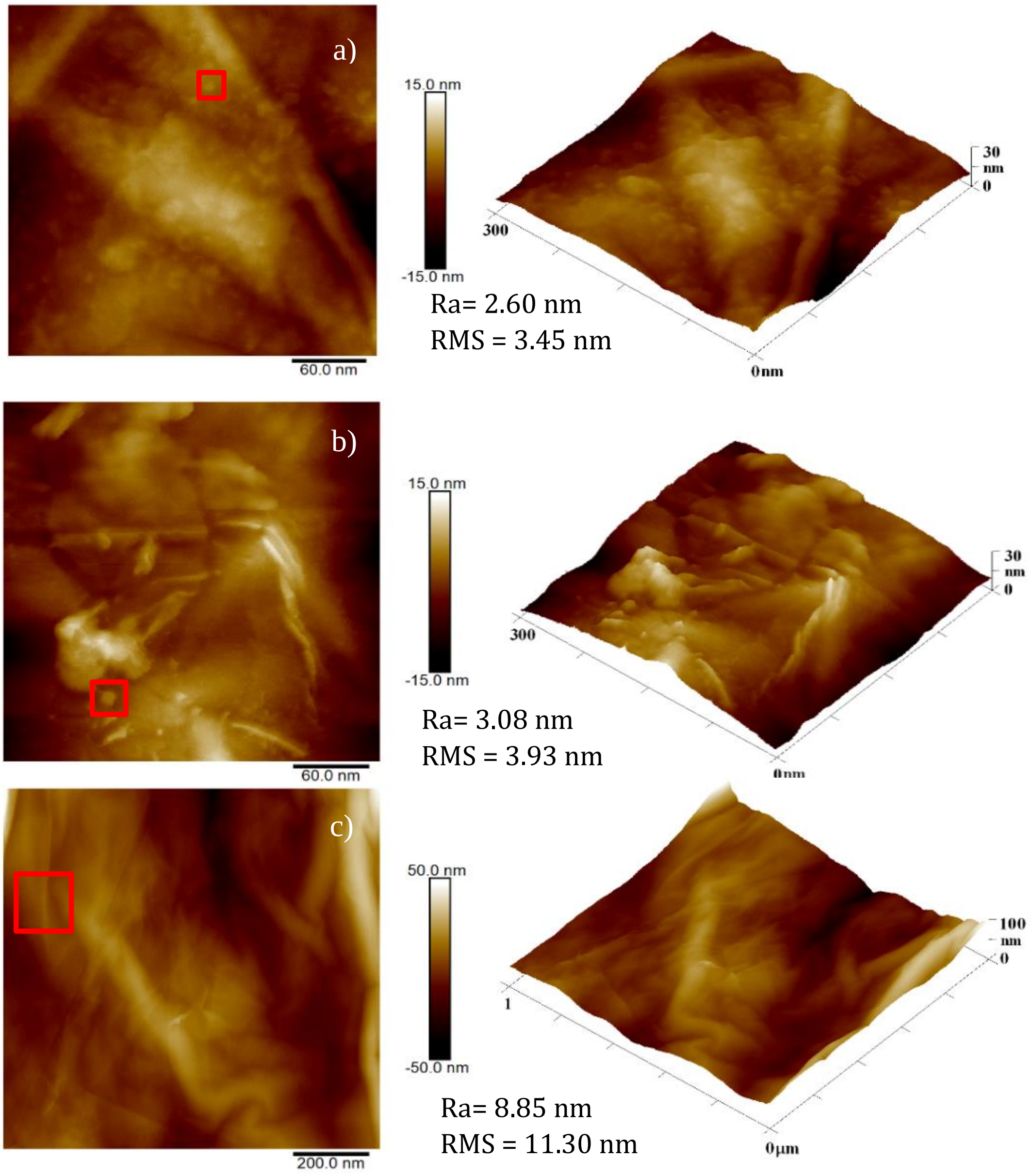

Figure S4. Two (to the left) and three(to the right) dimensional AFM images of GO irradiated by the 3 MeV H^+ ions at the $5x10^{11}$ ions/cm^2 a), at the $5x10^{12}$ ions/cm^2 b) and at the $5x10^{13}$ions/cm^2 c) fluences. In three (to the right) dimensional AFM images the average roughness (R_a) and the Root Mean Square (RMS) of the measured surface values are also indicated. (A colour version of this figure can be viewed online).

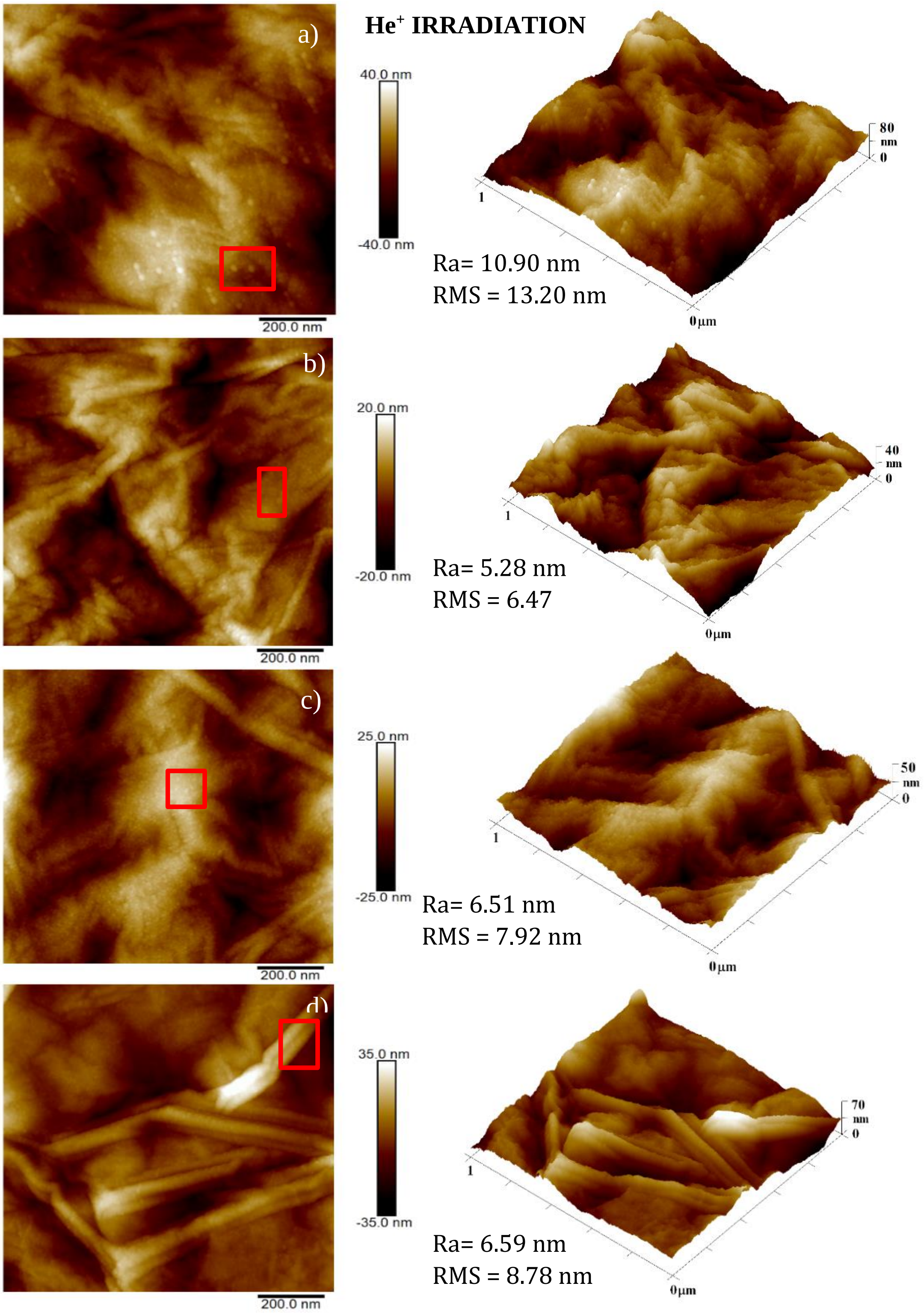

Figure S5. Two-dimensional AFM images of GO irradiated by the 3 MeV He^{+} ions at the $5x10^{11}$ ions/cm^2 a), $5x10^{12}$ ions/cm^2 b), $5x10^{13}$ions/cm^2 c) and $5x10^{14}$ ions/cm^2 d) fluences. In three dimensional (to the right) AFM images the average roughness (R_a) and the Root Mean Square (RMS) of the measured surface values are also indicated. (A colour version of this figure can be viewed online).

The decrease of the Ra and RMS values for O^+ ions irradiating the GO foils at $5x10^{13}$ ions/cm^2 is consistent with the sputtering yield for the oxygen ions in GO, deduced by the SRIM (Stopping and Range of Ions in Matter) software package.

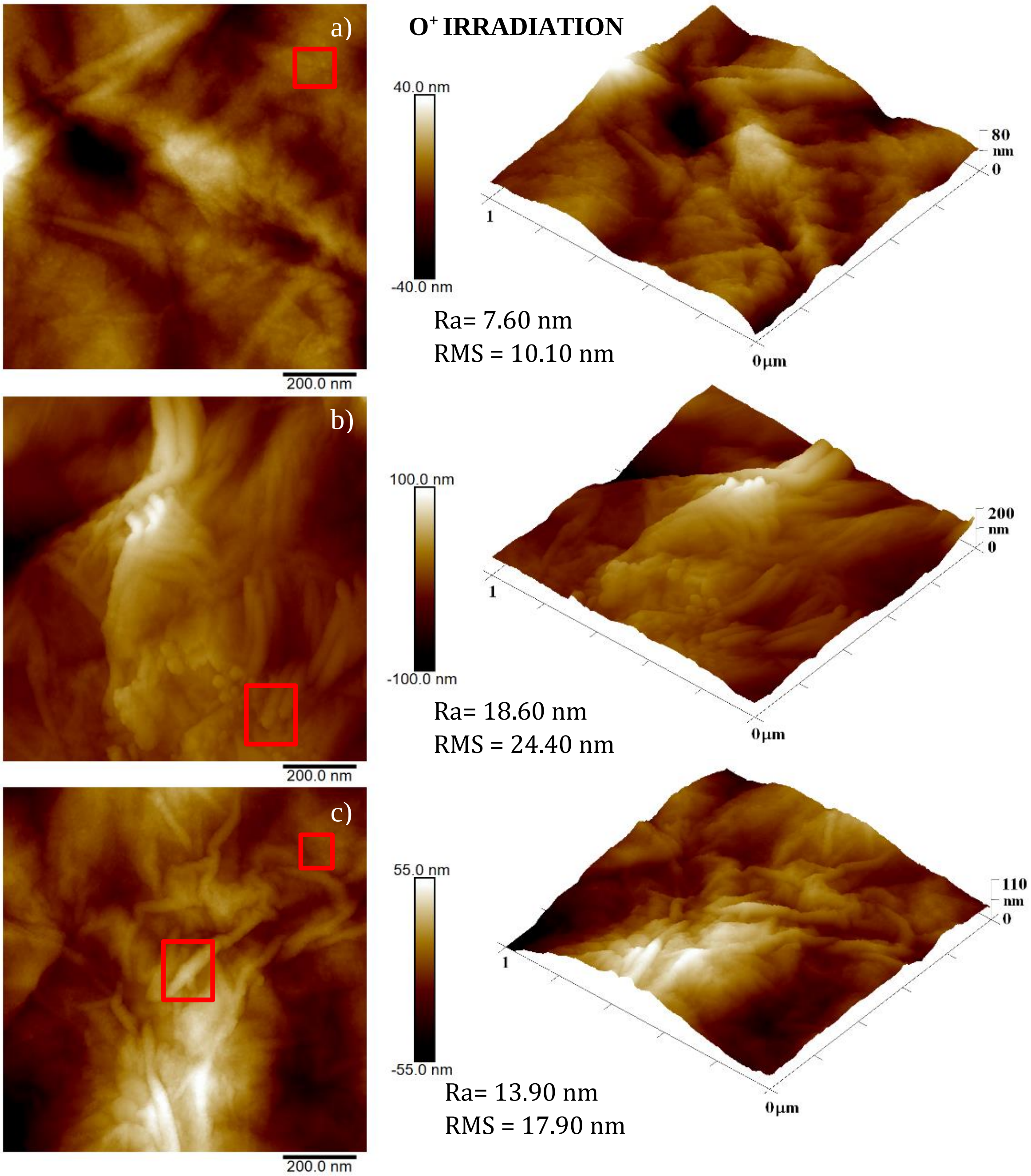

Figure S6. Two-dimensional AFM images of GO irradiated by the 3 MeV O^+ ions at the $5x10^{11}$ ions/cm^2 a), $5x10^{12}$ ions/cm^2 b), $5x10^{13}$ions/cm^2 c) fluences. In three dimensional AFM images (to the right) the average roughness (R_a) and the Root Mean Square (RMS) of the measured surface values are also indicated. (A colour version of this figure can be viewed online).

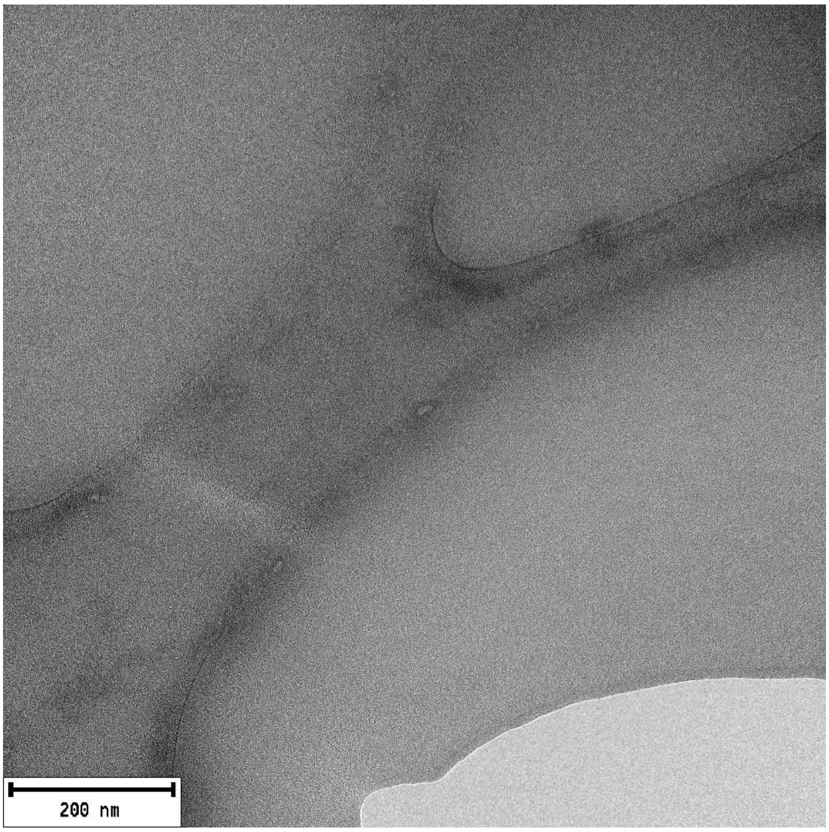

Figure S7. TEM micrograph of virgin GO.
